# Modulators of Hepatic Lipoprotein Metabolism Identified in a Search for Small-Molecule Inducers of *Tribbles Pseudokinase 1* Expression

**DOI:** 10.1371/journal.pone.0120295

**Published:** 2015-03-26

**Authors:** Marek M. Nagiec, Adam P. Skepner, Joseph Negri, Michelle Eichhorn, Nicolas Kuperwasser, Eamon Comer, Giovanni Muncipinto, Aravind Subramanian, Clary Clish, Kiran Musunuru, Jeremy R. Duvall, Michael Foley, Jose R. Perez, Michelle A. J. Palmer

**Affiliations:** 1 Therapeutics Platform, Center for the Science of Therapeutics, Broad Institute of MIT and Harvard, Cambridge, Massachusetts, United States of America; 2 Cancer Program, Broad Institute of MIT and Harvard, Cambridge, Massachusetts, United States of America; 3 Metabolite Profiling Platform, Broad Institute of MIT and Harvard, Cambridge, Massachusetts, United States of America; 4 Department of Stem Cell and Regenerative Biology, Harvard University, Cambridge, Massachusetts, United States of America; Harvard Medical School, UNITED STATES

## Abstract

Recent genome wide association studies have linked tribbles pseudokinase 1 (*TRIB1*) to the risk of coronary artery disease (CAD). Based on the observations that increased expression of *TRIB1* reduces secretion of VLDL and is associated with lower plasma levels of LDL cholesterol and triglycerides, higher plasma levels of HDL cholesterol and reduced risk for myocardial infarction, we carried out a high throughput phenotypic screen based on quantitative RT-PCR assay to identify compounds that induce *TRIB1* expression in human HepG2 hepatoma cells. In a screen of a collection of diversity-oriented synthesis (DOS)-derived compounds, we identified a series of benzofuran-based compounds that upregulate *TRIB1* expression and phenocopy the effects of *TRIB1* cDNA overexpression, as they inhibit triglyceride synthesis and apoB secretion in cells. In addition, the compounds downregulate expression of *MTTP* and *APOC3*, key components of the lipoprotein assembly pathway. However, CRISPR-Cas9 induced chromosomal disruption of the *TRIB1* locus in HepG2 cells, while confirming its regulatory role in lipoprotein metabolism, demonstrated that the effects of benzofurans persist in *TRIB1*-null cells indicating that TRIB1 is sufficient but not necessary to transmit the effects of the drug. Remarkably, active benzofurans, as well as natural products capable of *TRIB1* upregulation, also modulate hepatic cell cholesterol metabolism by elevating the expression of *LDLR* transcript and LDL receptor protein, while reducing the levels of *PCSK9* transcript and secreted PCSK9 protein and stimulating LDL uptake. The effects of benzofurans are not masked by cholesterol depletion and are independent of the SREBP-2 regulatory circuit, indicating that these compounds represent a novel class of chemically tractable small-molecule modulators that shift cellular lipoprotein metabolism in HepG2 cells from lipogenesis to scavenging.

## Introduction

Despite widespread use of cholesterol-lowering drugs, cardiovascular disease remains one of the leading causes of death worldwide and there is a need for novel approaches to improve therapies [1]. Epidemiological studies have repeatedly demonstrated that elevated levels of circulating LDL cholesterol (LDL-C) and triglyceride (TG)-rich remnant lipoproteins have strong associations with the development of coronary artery disease (CAD) and myocardial infarction (MI) [2–4]. Because 70% of LDL is removed from the circulation by LDL receptor-mediated uptake in the liver, therapeutic strategies that lead to elevated hepatic expression of the LDL receptor gene, *LDLR*, have proven to be efficacious in lowering LDL-C and provide protection from cardiovascular disease. Statins, through the inhibition of HMG CoA reductase, deplete cholesterol in the ER of hepatic cells, leading to the activation of the SREBP-2-dependent transcriptional program, which includes increased expression of LDLR. Paradoxically, clinical efficacy of statin therapy is limited by the fact that activation of SREBP-2 also leads to increased expression of proprotein convertase subtilisin/kexin type 9 (PCSK9), which acts as a negative regulator of LDL uptake by promoting degradation of LDL receptor. Recent results from clinical trials with anti-PCSK9 monoclonal antibodies suggest that PCSK9 blockade may indeed provide a more efficacious mechanism of elevating LDL receptor levels than traditional inhibition of HMG CoA reductase [5–8]. Alternative strategies to lower circulating LDL-C include treatments that lower hepatic secretion of very-low-density lipoprotein (VLDL) particles, the precursors of LDL particles, into the bloodstream. Recently introduced examples of such treatments include inhibitors of microsomal triglyceride transfer protein (MTP) and antisense DNA directed against apoB [9, 10]. Reduction of VLDL secretion leads to reduction of plasma TG, which is an independent cardiovascular disease risk factor, and its reduction brings benefits beyond lowering LDL-C alone [3, 4, 11]. Limitations and the side effects of statins as well as the side effects—particularly hepatic fat accumulation and liver toxicity—associated with apoB-directed treatments underscore the critical need for development of new therapeutic strategies to lower LDL-C and TG to prevent MI.

Recent genome-wide association studies (GWAS) have uncovered novel genes associated with CAD and MI and suggest novel approaches to developing improved therapeutics. *TRIB1* emerged in several GWAS as a novel cardiovascular locus, where the protective allele is strongly associated with decreased levels of circulating LDL-C and triglycerides (TG), increased levels of high-density lipoprotein (HDL) as well as with reduced incidence of CAD and MI [12, 13]. Additional studies in mice confirmed the link between *Trib1* and lipid levels and demonstrated that increased expression of *Trib1* is protective against the disease [14]. Hepatic overexpression of *Trib1* in mice reduced the secretion of VLDL particles from the liver into the bloodstream and, consistent with this observation, overexpression of *TRIB1* in human hepatoma cells reduced apoB secretion. The precise molecular mechanism by which overexpression of *Trib1* regulates the rate of VLDL particle formation and secretion is not known, although hepatic overexpression of *Trib1* in mice correlates with decreased expression of TG biosynthetic genes (*Fasn*, *Scd1*, *Dgat2*) and decreased rate of TG formation, suggesting that reduced TG availability leads to insufficient apoB lipidation, targeting nascent apoB to ER-associated degradation [14].

TRIB1, as other members of tribbles family, is a pseudokinase and is thought to act as an adaptor protein in the MEK/ERK signaling pathways [15]. It regulates a wide array of cellular processes, ranging from cell migration in macrophages and in smooth muscle cells [16], through macrophage polarization [17] and myeloid leukemogenesis [18], to regulation of lipid metabolism in hepatocytes [14]. In addition to plasma lipid levels, the protective *TRIB1* allele has been also linked to lower blood levels of liver enzymes, reduced risk of non-alcoholic fatty liver disease and to longer sleep [13, 19, 20]. SNPs leading to upregulation of *TRIB1* likely have very selective effects that are restricted to one gene and it is unlikely that such selectivity could be achieved with drugs. Nonetheless identification of small-molecule upregulators of *TRIB1* could potentially open up a path to identification of novel modulators of lipid metabolism and provide new tools for studying TRIB1 regulation. To this end we developed a qRT-PCR screen to identify compounds that can upregulate *TRIB1* expression. We chose to screen the Broad Institute small-molecule library that includes 100,000 novel compounds derived from diversity-oriented synthesis (DOS), a synthetic strategy to access complex and diverse compounds in an efficient manner [21–24]. The DOS compounds are enriched in sp^3^ carbons and chiral centers leading to more 3-dimensionality compared to flat, achiral compounds often found in commercial libraries. In addition, the DOS compound collection is designed to elucidate initial stereochemical and appendage structure-activity relationships (SAR) from primary and secondary screens [25, 26]. Herein, we describe the identification of BRD0418, a DOS molecule that regulates *TRIB1* expression. Characterization of BRD0418 revealed a broader profile of gene-expression changes that lead to decreased rate of VLDL production and increased rate of LDL uptake in cells of hepatic origin. This data indicate that treatment with BRD0418 leads to reprogramming of hepatic lipoprotein metabolism from lipogenesis to scavenging.

## Materials and Methods

### Cell culture and chemicals

HepG2 cells (ATCC) were maintained in Growth medium—DMEM High glucose with sodium pyruvate and glutamine (Invitrogen), 10% FBS (Hyclone), Penicillin (100 units/mL), Streptomycin (100 μg/mL) and glutamine (2 mM) (Invitrogen). HepG2 cells were incubated at 37°C, 5% CO_2_. For cholesterol depletion experiments cells were grown in the DMEM media containing the indicated concentration of lipoprotein deficient serum (LPDS, Sigma). Oligomycin A (Sigma), 9-cis-retinoic acid (Enzo), ATRA (Sigma), Berberine (Sigma), CP-775146 (Tocris), fenofibrate (Tocris), fexaramine (Tocris) PGJ2 (Cayman), AICAR (Sigma) and the compounds from the screening collection were prepared as 10 mM solutions in DMSO, diluted appropriately in DMSO and delivered to cell culture at indicated concentrations keeping the concentration of vehicle constant at 0.25%. Oncostatin M was obtained from R&D Systems.

### Synthesis of BRD0418

Detailed description of the synthesis of BRD0418 is provided in Supplemental Information (S1 Protocol. Synthesis of BRD0418).

### Compound screening and generation of cDNA

HepG2 cells were plated in white 384 well plates (Corning) in 40 μL of growth media and incubated overnight. The next day 100 nanoliters of compound was transferred to the plates and incubated overnight. Each plate contained 32 wells treated with DMSO to statistically empower hit calling. After 18–22 hr of incubation RNA was harvested and cDNA was synthesized using the Ambion Cells-to-CT reagents (Life Technologies). Specifically, the cells were washed 2x with PBS and then lysed with 10 μL of Cells-to-CT Lysis with DNase reagent and incubated at room temperature for 10 minutes. After the incubation 1 μL of Stop solution was added. Two μL of the RNA lysis was added to 8 μL of Cells-to-CT cDNA reagent in a 384 well plate (Axygen) and incubated as follows: 37°C for 1 hr, 95°C for 5 min. then 4°C until frozen at-80°C.

### Gene expression analysis by qRT-PCR

Gene expression was measured by quantitative PCR in a 5 μL reaction in a 384 well plate (Roche) comprising 2.5 μL Probes Master Mix (Roche) 0.125 μL of human target gene primer/probe, 0.125 μL of human calibrator gene primer/probe (*GAPDH* or *B2M*), 1.25 μL of PCR grade water and 1 μl of cDNA. The identities and sources of the primer/probe sets used are listed in Table 1. Thermocycling was performed in a Roche Light Cycler 480 II instrument using the following conditions: 95°C for 10 minutes, 95°C for 10s, 60°C for 30s for 55 cycles then 40°C for 30s. Relative gene expression was calculated using the equation 2^-ΔΔCT^ [27]. For screening the hits were called for individual plates. Compounds that stimulated >2-fold upregulation of *TRIB1* over GAPDH expression and had the ΔΔCt ((*TRIB1*_DMSO_ Ct—*GAPDH*_DMSO_ Ct)-(*TRIB*_Cpd_ Ct—*GAPDH*_Cpd_ Ct)) Z-score <-2 and GAPDH Ct Z-score between 10 and-10 were considered hits. The Z-scores were calculated using standard deviations obtained for 32 DMSO treated wells present in each plate.

**Table 1:** qRT-PCR primer/probes.

Gene	Assay ID/Sequence	Source
TRIB1	Hs00921832_m1	Applied Biosystems
GAPDH	4326317E	Applied Biosystems
B2M	4310886E	Applied Biosystems
PCSK9	Hs.PT.49a.3004786	IDT
LDLR	Hs.PT.49a.20193252	IDT
SCD-1	Hs.PT.49a.20912150	IDT
FASN	Hs.PT.49a.20033464.g	IDT
HMGCR	Hs.PT.49a.1212149.g	IDT
HMGCS1	Hs.PT.49a.19114256	IDT
MTTP	Hs00165177_m1	Applied Biosystems
APOC3	Hs00163644_m1	Applied Biosystems
TRIB1	ACCAAGGCCTATGTCTTCTTTG GCGGAGACAATCTGCTTGAA TTCGCACATAGGAGTGCATGTCCC	IDT custom

### Luminex transcriptional profiling

The expression profiling of 1000 landmark genes selected based on potential to infer expression of remaining gene transcripts [28] was carried out using bead-based detection assay (Luminex, Austin, TX) essentially as described [29]. Briefly, the HepG2 cells were treated with compounds and the cell lysates were obtained as described in the *Compound screening* section above. The first strand of cDNA was synthesized from mRNA captured in oligo-dT coated turbocapture plates (Qiagen) using M-MLV reverse transcriptase and the selected cDNAs were amplified using gene specific probes by ligation dependent amplification (LDA) followed by detection using Luminex beads conjugated with gene specific oligonucleotide tags [29].

### ELISA assays

HepG2 cells were plated in black clear bottom (Costar) 96-well plates at 40,000 cells/well in 100 μL of growth media. On Day 2 media was changed and cells were treated with compounds for 24 hr. After 24 hr medium was removed and used for the PCSK9 (R&D Systems) and apoB (MAb Tech) ELISAs. For the apoB and PCSK9 ELISAs 50 μL and 10 μL of medium were used, respectfully. For the LDLR ELISA (R&D Systems), cells were washed once with PBS (Invitrogen), lysed with 100 μL PBS/0.1% Igepal (Sigma) and frozen at-80°C until analyzed. Ten μL of lysate was used for the analysis.

### Transferrin AlphaLISA assay

On Day 0 HepG2 cells were plated in a white 384 well plate (Corning) at 10,000 cells/well in 30 μL of growth media at 37°C with 5% CO_2_. On Day 1 the media was removed and the cells washed with 100 μL of PBS using a Biotech plate washer. Thirty μL of DMEM media containing 0.5% FBS was then added to the cells. Cells were treated with 100 nL of compound. Brefeldin A (1 μM) was used as a positive control for inhibition of Transferrin secretion. On Day 2 the Transferrin AlphaLISA (Perkin Elmer) was performed per the manufacturer’s protocol.

### Cell viability assay

HepG2 cells were plated in white 384 well plates (Corning) at 2000 cells/well in growth medium and incubated overnight at 37°C with 5% CO_2_. The next day cells were treated with 100 nL of compound for 24 hr. Cellular ATP levels were measured using CellTiter-Glo (Promega). For testing effects of compounds on mitochondria-dependent oxidative energy production cells were grown in media containing 10 mM galactose instead of 25 mM glucose as main carbon source [30].

### Stable isotope labeling and analysis of TGs in HepG2 cells

The cells were plated at 40,000 cells/well in 96-well plate in growth media, incubated overnight and then treated for 24 hours with compounds as indicated. For stable isotope labeling of TG the cells were washed with PBS, incubated for at least 15 minutes with DMEM supplemented with 25 mM HEPES (pH 7.5) containing 0.1% FAF-BSA and then after media replacement incubated at 37°C with DMEM containing 25 mM HEPES (pH 7.5), 0.1% FAF-BSA and 20 μM ^13^C_3_-D_5_-glycerol for indicated periods of time. The lipids were extracted after the media removal by incubating the cells with 150 μl isopropanol at room temperature for 15 min with shaking. The cell extracts (120 μl) were cleared by centrifugation and concentrated 5-fold through drying and resuspension in isopropanol. Liquid chromatography-tandem mass spectrometry (LC-MS) analyses of cell extracts were conducted using an Open Accela 1250 U-HPLC coupled to a Q Exactive hybrid quadrupole orbitrap mass spectrometer (Thermo Fisher Scientific; Waltham, MA). Extracts (10 μL) were injected directly onto a 150 x 3.0 mm Prosphere HP C4 column (Grace, Columbia, MD). The column was eluted isocratically at a flow rate of 350 μL/min with 80% mobile phase A (95:5:0.1 vol/vol/vol 10 mM ammonium acetate/methanol/acetic acid) for 2 minutes followed by a linear gradient to 80% mobile phase B (99.9:0.1 vol/vol methanol/acetic acid) over 1 minute, a linear gradient to 100% mobile phase B over 12 minutes, and then 10 minutes at 100% mobile-phase B. MS analyses were conducted using electrospray ionization in the positive ion mode using full scan analysis at 70,000 resolution and 3 Hz data acquisition rate. Monoisotopic and ^13^C_3_-D_5_-TAG LC-MS peaks were integrated using TraceFinder 2.0 (Thermo Fisher Scientific; Waltham, MA).

### LDL uptake

HepG2 cells were plated at 3000 cells/well in a 384 well plate (Corning) in growth medium. After an overnight incubation 10 μL of 5x compound was added and the plates were incubated overnight. The cells were then washed with phenol red free DMEM (Invitrogen) and incubated with 30 μl of BODIPY-FL-LDL (Invitrogen) diluted to final concentration 5 μg protein/mL in phenol red free DMEM for 0.1 to 5 hr. LDL uptake was stopped by fixing the cells with 3% formaldehyde. Nuclei were stained with Hoechst 33342 diluted in PBS for 10 minutes. The cells were then washed with PBS and imaged using an IXM microscope. Image analysis and quantification was performed with MetaXpress software (ver. 3.1.0.97, Molecular Devices) using the Multi Wavelength Cell Scoring application module.

### Western blot analysis

HepG2 cells were seeded in 6-well plates at approximately 800,000 cells /well, incubated in growth media at 37°C for 24 hours, further incubated for 20 hours in serum-free DMEM and then treated with EGF (100 ng/mL), OSM (100 ng/mL) or BRD0418 (10 μM) for the indicated times. At the end of treatment, cells were washed with PBS and lysed in RIPA lysis buffer (50mM Tris-HCl, 1% IGEPAL, 0.5% Na-deoxycholate, 0.1% SDS, 150 nM NaCl, 2 mM EDTA, 50 mM NaF) with protease inhibitor cocktail (Roche). Lysates were incubated on ice for 1 hour with frequent agitation, centrifuged at 13,000 rpm for 15 minutes at 4°C and then equal amount of protein from each lysate (∼ 50 μg) was denatured, subjected to electrophoresis using NuPAGE Novex 4–12% Bis-Tris Gels and transferred to PVDF membranes with iBlot Gel Transfer Device (Invitrogen) according to manufacturer’s instructions. The ERK1/2 phosphorylation was detected using rabbit polyclonal antibodies directed against p44/42 MAPK (Erk1/2) and Phospho-p44/42 MAPK (Erk1/2) (Cell Signaling Technology) and the goat anti-rabbit IgG (H+L) secondary antibodies conjugated with HRP (Thermo Scientific). After standard incubations and washing the membranes were exposed with SuperSignal West Femto Maximum Sensitivity Substrate (Thermo Scientific) and band intensities were calculated using Image J software.

### *TRIB1* vector transfection

HepG2 cells (600,000 cells/well) were plated in a 6 well plate (Corning) in growth medium overnight at 37°C with 5% CO_2_. A *TRIB1* expression vector (Origene) was transfected using Fugene 6 (Roche) in 1 mL of Opti-MEM (Invitrogen) for 6 hr. After 6 hr, 2 mL of growth medium was added to the wells and incubated overnight. After the overnight incubation the media was changed to growth media and supplemented with 500 μg/mL Geneticin (Invitrogen). After 10 days, Geneticin-resistant clones were transferred to a 96-well plate (Costar) for expansion. After expansion into a 48 well plate (Corning) clones were analyzed for *TRIB1* mRNA expression by qRT-PCR.

### CRISPR-Cas9 induced disruption of chromosomal *TRIB1* locus

HepG2 cells were plated in a 6 well plate (400,000 cells/well) in growth medium and after overnight incubation they were transfected using X-tremeGENE HP DNA transfection reagent (Roche) with 1.5 μg CRISPR-Cas9 plasmid (pCMV-Cas9-GFP, Sigma) and 1.5 μg of the repair template DNA fragment. The CRISPR-Cas9 plasmid carried the target sequence 5’- GGATACACGCTTCGGCCTTCTC-3’ in the guide RNA gene. The repair template consisted of three segments that were individually PCR amplified using HepG2 genomic DNA as a template for the *TRIB1* target flanking segments and pCINeo plasmid as a template for Neo^R^ cassette segment. The sequences of the PCR primers were designed to provide an overlap with the adjacent segments to allow for final PCR extension and amplification of the entire repair template. The sequences of PCR primers are listed in Table 2. All PCR amplification reactions were carried using Q5 High-Fidelity DNA Polymerase (New England Biolabs) and a touchdown amplification protocol. The repair template was cut with XhoI and SmaI and cloned in the pFN28A vector (Promega) cut with XhoI and PmeI. Gel purified XhoI/BbvI 2991 bp restriction fragment released from this plasmid was used for HepG2 transfection. Next day after transfection the media was replaced and supplemented with 500 μg/mL Geneticin (Invitrogen). Geneticin resistant clones were isolated and expanded after 3 weeks of selection. Clones that carry the designed chromosomal disruption were identified using diagnostic PCR reactions as described in the Results section. The sequences of the diagnostic primers T_U_, T_D_, N_U_ and N_D_ are listed in Table 2. Genomic DNA for PCR based screening was prepared using QuickExtract reagent (Epicentre) from cells grown in 96-well plate.

**Table 2:** PCR primers used for HepG2 *ΔTRIB1*::*Neo* ^*R*^ clone construction.

Primer	Sequence (5’-> 3’)
TRIB1LXhoI	ATATCTCGAGATTCCATACCCTTCCTGTGAGAATTGTAAC
TRIB1LAsiSI(Neo)	GTTATTTCAGGCCATGGTGCTGCGCGATCGCAGTGCATGTCCCCAAAGTCCTTCTC
NeoAsiSI(TRIB)	GAGAAGGACTTTGGGGACATGCACTGCGATCGCGCAGCACCATGGCCTGAAATAAC
NeoMfeI(TRIB)	GGCGGCTTCCTCTTCCCGCAGCAATTGCACACAAAAAACCAACACACAGATG
TRIB1RMfeI(Neo)	CATCTGTGTGTTGGTTTTTTGTGTGCAATTGCTGCGGGAAGAGGAAGCCGCC
TRIB1RSmaI	ATATCCCGGGAAACCTCAGCTTTGAATCTTAATGCCTC
T_U_	TGTCACCGTTTCCAGTTGAGT
T_D_	TGCGAGGTCAGATACAAAGTCA
N_U_	GTTGCTGACTAATTGAGATGCATGC
N_D_	CTATCGCCTTCTTGACGAGTTCTTCTG

## Results

### High-throughput chemical screen

**Fig 1:**
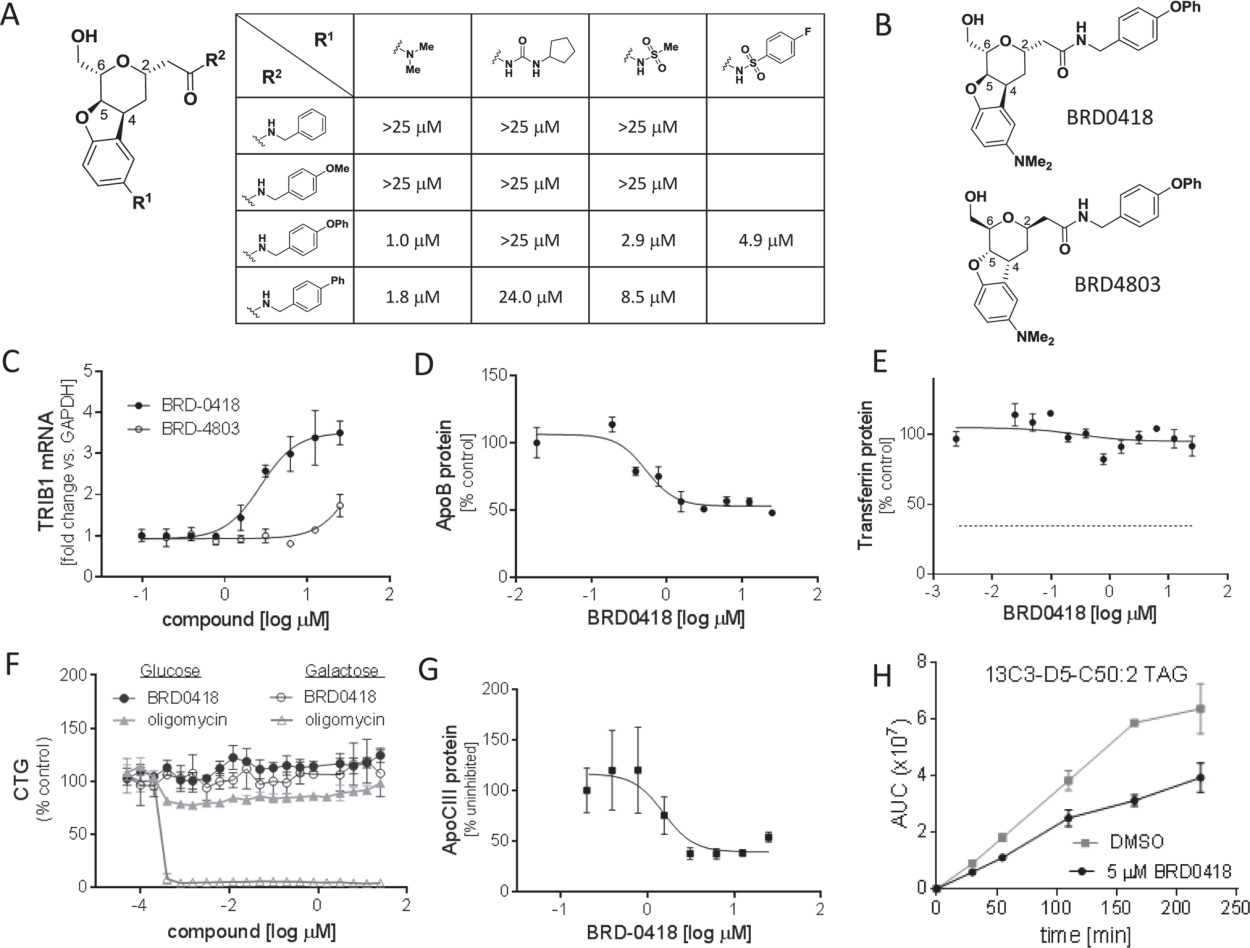
A qRT-PCR screen identifies a series of benzofuran scaffold based compounds that mimic effects of *TRIB1* overexpression in HepG2 cells. (*A*) Activity of the screening hit BRD0418 and its structural analogs in the *TRIB1* upregulation assay. Numbers in the table represent EC_50_ values obtained in a multiple-dose confirmation assay in cells treated with compounds for 6 hours. (*B*) Chemical structure of BRD0418 and its enantiomer BRD4803. (*C*) Dose dependent upregulation of *TRIB1* transcript relative to *GAPDH* calibrator control in HepG2 cells treated with BRD0418 and BRD4803 for 6 hours. Data represent mean fold change **±** S.E. (*error bars*) of three independent replicates. The data were fitted using GraphPad Prism software. (*D*) BRD0418 inhibits secretion of ApoB from HepG2. The levels of apoB in the media 24 hours after the treatment with indicated concentrations of the compound were measured by ELISA as described in Experimental Procedures. Data represent mean **±** S.E. (*error bars*) of three replicates. (*E*) BRD0418 had no effect on secretion of transferrin. The levels of transferrin in the media 24 hours after the treatment with indicated concentrations of the compound were measured by ELISA as described in Experimental Procedures. The inhibition level of transferrin secretion by 6 μM brefeldin A is indicated by dashed line. (*F*) Treatment with BRD0418 had no effect on the cellular level of ATP in HepG2 cells. The cells were grown either on glucose or on galactose media, treated with a series of doses of BRD0418 or Oligomycin A for 24 hours and the cellular ATP levels were measured by the Cell-Titer-Glo assay. (*G*) BRD0418 inhibits secretion of ApoCIII from HepG2 cells. ApoCIII protein levels were measured in the media by ELISA 20 hours after compound treatment. Data were normalized to lowest dose value and represent mean ± S.E. (*error bars*) of three replicates. (*H*) Inhibition of triglycerides formation in HepG2 cells. The cells were pre-incubated with 5 μM BRD0418 for 24 hours and then incubated with the stable isotope labeled glycerol (^13^C_3_-D_5_-glycerol) for varying amounts of time. The incorporation of the ^13^C_3_-D_5_-glycerol into triglycerides was quantified in the lipid extracts by high resolution mass spectroscopy. The plot shows incorporation of ^13^C_3_-D_5_-glycerol into one of the species of triacylglycerol, ^13^C_3_-D_5_-C50:2 TAG. Data represent mean area under the curve (AUC) values **±** S.D. (*error bars*) for two replicates.

Building on the observations that linked the increased expression of the *TRIB1* gene to improved lipid profile and reduced incidence of CAD and MI, we developed a quantitative RT-PCR-based phenotypic assay to identify compounds that upregulate *TRIB1* expression in HepG2 human hepatocellular carcinoma cells. We performed a high-throughput screen using the Broad small-molecule library, which includes 100,000 DOS compounds, testing each compound in duplicate at 12 μM concentration and looking for upregulation of *TRIB1* expression in HepG2 cells. We focused on compounds inducing *TRIB1* >2-fold relative to *GAPDH* and compared to DMSO controls, selecting only compounds with no significant effect on the reference gene expression. Initial hits were retested in a dose-response format in two hepatoma cell lines (HepG2 and HuH7) using a different reference gene (*B2M*) and screened for effects on viability as measured by cellular ATP levels. Confirmed hits with no cell toxicity were further tested for effects on apoB secretion in HepG2 cells. After completing the triaging process and reviewing existing SAR generated from the DOS collection, we identified a benzofuran scaffold [24] (Fig. 1A), which upregulated *TRIB1* in both cell lines and was able to reduce apoB secretion in HepG2 cells >30%, thus mimicking the effects of *TRIB1* cDNA overexpression [14]. Initial SAR from the benzofuran library (Fig. 1A) gave us confidence that the observed effect was due to the small molecule and not to an artifact or impurity. Moreover, the enantiomer of BRD0418 was largely inactive suggesting that biological effects of BRD0418 result from selective and specific interactions with a cellular target (Fig. 1B, C). Changes at the aniline site (R^1^) were mostly tolerated, with both the tertiary amine and sulfonamide showing activity and therefore indicating that none of the particular properties of neither of those substituents is important for producing the phenotype. The amide building block (R^2^) showed less tolerability to change, as bulky substituents off of the benzyl amine were required for increased potency. Based on the observed potency, we selected BRD0418 (Fig. 1B) for more detailed characterization.

### BRD0418 phenocopies the effects of *TRIB1* overexpression

Overexpression of *TRIB1* cDNA in HepG2 cells reduced the rate of triglyceride synthesis and downregulated apoB secretion, presumably due to insufficient lipidation of apoB, which leads to targeting of apoB for ER-associated degradation (ERAD) [14]. Treatment of HepG2 cells with BRD0418 for 6 hours resulted on average in 3.5-fold up-regulation of *TRIB1* transcript levels, with half-maximal effect achieved at 2.3 μM concentration of the compound (Fig. 1C). BRD0418 inhibited secretion of apoB from HepG2 cells with similar potency, reducing by about 50% the level of apoB100 present in the media over the cells treated with the compound for 24 hours (Fig. 1D). The secretion of transferrin was not affected by BRD0418 treatment, indicating that the compound did not have a general effect on secretion (Fig. 1E). In the same range of doses, BRD0418 had no effect on cell viability as measured by cellular ATP levels (Fig. 1F). Oligomycin A, an inhibitor of OXPHOS at the mitochondrial ATP synthase level, was recently shown to induce *TRIB1* mRNA in HepG2 cells through a MAPK dependent mechanism [31]. Since immortalized and highly proliferative cells lines, like HepG2, tend to synthesize majority of its ATP from glycolysis the cellular ATP levels in cells grown in standard glucose-containing media do not change as result of drug induced mitochondrial defects [30]. Therefore in order to determine if benzofurans might be acting through a similar mechanism we also measured the effects of BRD0418 on cellular ATP levels in cells grown in galactose-containing media, conditions that force cellular ATP production through mitochondrial oxidative phosphorylation. The ATP levels for cells grown in the presence of oligomycin on glucose were inhibited no more than 21%, whereas for cells grown on galactose they were inhibited more than 95%. Treatment with BRD0418 did not affect ATP levels in cells grown under either condition (Fig. 1F) suggesting that it does not act as mitochondrial stressor. Next, we examined the effect of BRD0418 on the rate of triglyceride synthesis by measuring the incorporation of stable isotope-labeled glycerol into triglycerides over 4 hours in cells preincubated for 24 hours with 5 μM compound. BRD0418 treatment significantly reduced formation of several species of triglycerides that were quantified in lipid extracts by mass spectrometry (Fig. 1H, and data not shown). These results indicate that the BRD0418-induced upregulation of *TRIB1* expression mimics the effects of *TRIB1* cDNA overexpression.

### BRD0418 broadly affects lipid metabolic genes

**Fig 2:**
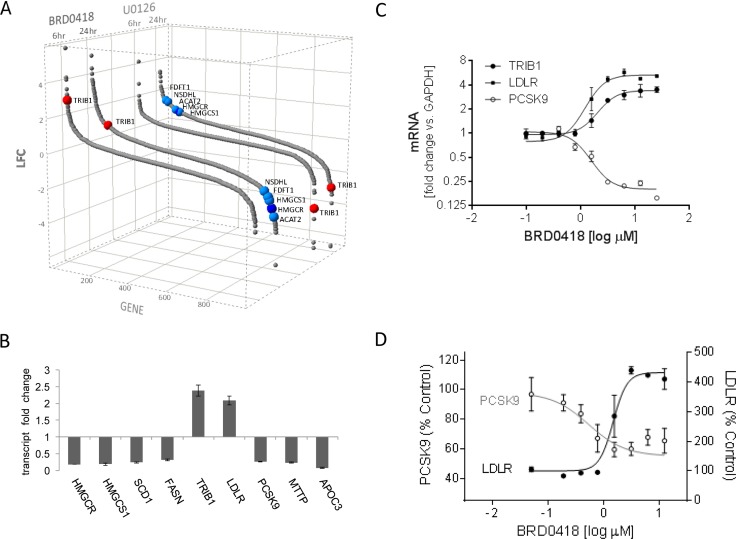
BRD0418 modulates expression of the key genes involved in cholesterol and triglyceride biosynthesis, VLDL production and in LDL uptake in HepG2 cells. (*A*) Summary of transcriptional profiling of cells treated with *TRIB1* inducer (BRD0418) and *TRIB1* inhibitor (U-0126) identified in the primary high-throughput chemical screen. Level of expression of 1000 landmark genes (L1000 approach) was measured using Luminex quantification. LFC is the average of the log_2_ fold change of gene expression level for triplicate treatment with 10 μM BRD0418 and for triplicate treatment with a 3.3 μM U0126 relative to DMSO control treatments. Profiles for 6 hour and 24 hour compound treatments are depicted. For clarity only *TRIB1* (red) and lipid biosynthetic genes (blue) are indicated. (*B*) Changes in transcript levels for indicated lipid metabolic genes were measured by qRT-PCR in HepG2 cells treated for 22 hours with a single, 25 μM dose of BRD0418. The values are normalized to DMSO controls and represent means of two experiments **±** S.D. (*error bars*). (*C*) Changes in transcript levels in response to serially diluted doses of BRD0418 measured in HepG2 cells by qRT-PCR 6 hours (*LDLR* and *TRIB1*) and 24 hours (*PCSK9*) post treatment. Data represent mean fold change **±** S.E (*error bars*) of three replicates. (*D*) Levels of secreted PCSK9 protein and cellular LDLR protein were measured by ELISA 24 hours post treatment with BRD0418 in the media and in the cell lysates, respectively. Data represent mean **±** S.E. (*error bars*) for three replicates.

Early in the triage process, we performed an abbreviated transcriptional profiling of 1000 landmark genes (L1000) using Luminex detection [29] on HepG2 cells treated for 6 or 24 hours with selected screening hits. Evaluation of L1000 data not only confirmed by an alternative quantification methodology (Luminex LDA vs. qRT-PCR and quantile normalization vs. single reference gene transcript) the *TRIB1* upregulating activity of BRD0418 and other benzofuran hits, but also indicated that *TRIB1* upregulators modify gene expression more broadly affecting gene sets involved in signal transduction, in responses to external stimuli and in metabolism, including lipid metabolism (S1 Table. GO terms enrichment analysis). For example, expression of cholesterol biosynthetic genes *ACAT2*, *HMGCR*, *HMGCS1*, *FDFT* and *NSDHL* was inhibited more than 3-fold in benzofuran treated cells (Fig. 2A). The expression of *TRIB1* was more strongly elevated at 6 hours than at 24 hours, whereas the downregulation of cholesterol biosynthetic genes appeared to be secondary and was apparent only after the longer treatment.

To confirm and to extend the L1000 observations, we measured changes in several key lipid metabolic genes by qRT-PCR. A 24-hour treatment with 25 μM compound led to significant downregulation of cholesterol biosynthetic genes (*HMGCR*, *HMGCS*), fatty acid biosynthetic genes (*FASN*, *SCD1*) and genes involved in lipoprotein assembly (*MTTP*, *APOC3*) (Fig. 2B). The finding that BRD0418 reduces expression of *APOC3* is consistent with *TRIB1*-driven inhibition of lipoprotein assembly and apoB secretion, and was further confirmed in the dose-response experiments that showed potent downregulation of *APOC3* transcript (data not shown) and apoCIII protein secretion in HepG2 cells treated with BRD0418 (Fig. 1F). Notably, BRD0418 treatment led to decoupled changes in the expression of two SREBP-2 co-regulated genes directly involved in LDL uptake, namely to upregulation of *LDLR* and downregulation of *PCSK9* levels (Fig. 2B). The dose-response experiment demonstrated that the changes in expression of *LDLR* and *PCSK9*, measured 6 hours and 24 hours post-treatment, respectively, are produced by the similar dose of the compound as upregulation of *TRIB1* (Fig. 2C). Moreover, changes in gene expression were mirrored closely in changes in protein levels, leading to 4-fold increase of the LDL receptor in cells and to 2-fold decrease of secreted PCSK9 in the media (Fig. 2D).

**Fig 3:**
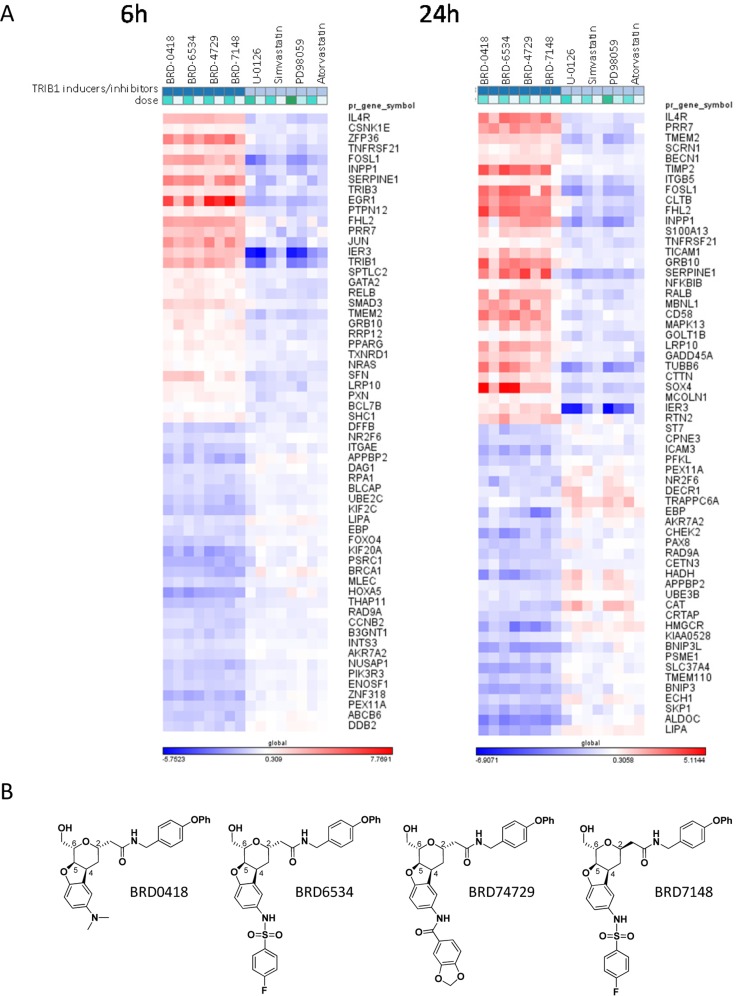
Marker selection of top genes differentially expressed in response to treatment with *TRIB1* expression inducers (BRD0418 and its active analogs) in comparison to treatments with *TRIB1* expression inhibitors, which included strong inhibitors (U0126 and PD-98059) and weak inhibitors (simvastatin and atorvastatin). (*A*) A heatmap of 60 genes showing strongest positive and negative difference in responses to two groups of treatments (inducers vs. inhibitors). Color scale represents logarithm fold change (LFC) values calculated relative to DMSO control treatments. The matrix analysis and visualization was carried out using GENE-E software (http://www.broadinstitute.org/cancer/software/GENE-E/index.html). (*B*) Chemical structures of *TRIB1* inducers from the benzofuran class profiled in the L1000 Luminex assay.

Interestingly, we found in the L1000 data analysis that the changes in *TRIB1* and cholesterol metabolism genes in HepG2 cells treated with BRD0418 were strongly inversely correlated with the responses to MEK inhibitors, U0126 and PD98059, which were included in the analysis since they were identified in the primary screen as inhibitors of *TRIB1* expression (Fig. 2A). The inverse correlation of transcriptional responses to benzofuran compounds and to MEK inhibitors was further highlighted by marker selection analysis, which ranks genes according to difference in response between the two groups (Fig. 3). The L1000 gene expression data showed that the response to benzofurans was rather broad and included a number of early response genes (e.g. *EGR1*, *JUN*, *IER3*, *ZFP36*, *SERPINE1*) suggesting activation of MAPK signaling pathways (Fig. 3).

### BRD0418 stimulates LDL uptake

**Fig 4:**
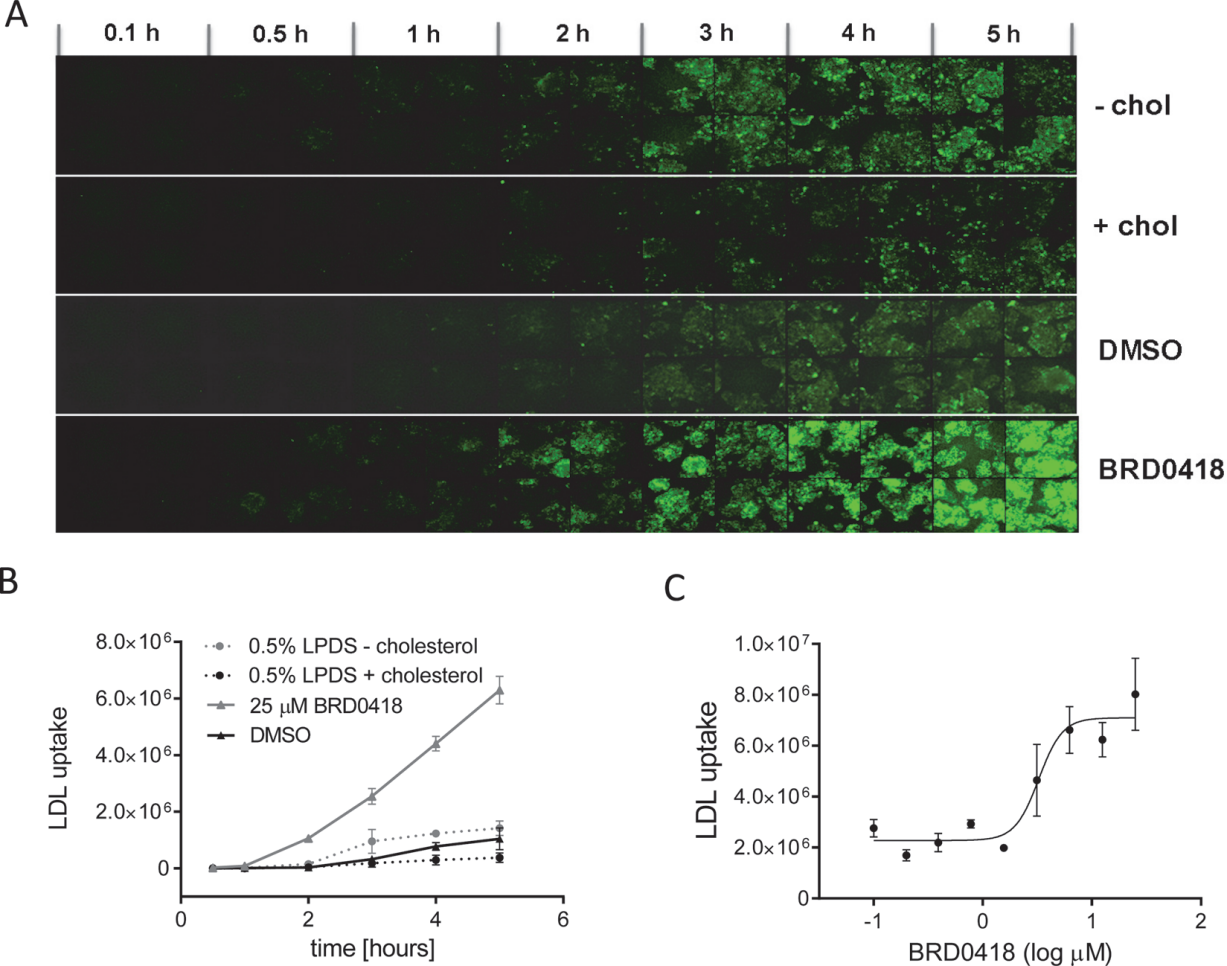
*TRIB1* inducers stimulate uptake of LDL in HepG2 cells. (*A*) Microphotographs of HepG2 cells stimulated with BRD0418 treatment or with cholesterol depletion in the presence of fluorescent dye labeled LDL. The cells grown under standard culture conditions (10% FBS) were treated for 20 hours with 25 μM BRD0418 and the uptake of BODIPY-FL conjugated LDL particles was monitored for 5 hours using high content microscopy. To control for regulation of LDL uptake by cholesterol the cells were grown in the media depleted for cholesterol (0.5% LPDS) or supplemented with cholesterol [(0.5% LPDS supplemented with cholesterol (10mg/mL cholesterol and 1mg/mL 25-OH cholesterol)]. (*B*) The uptake of BODIPY-FL-LDL by HepG2 cells depicted in panel A was quantified using the Multi Wavelength Cell Scoring algorithm provided in the MetaXpress software application (Molecular Devices). (*C*) Dose dependent effect of BRD0418 on LDL uptake. Cells were pretreated with the compound at the indicated concentration for 20 hours, incubated with BODIPY-FL-LDL for 5 hours and imaged by microscopy. The uptake was quantified using MetaXpress software and the data were fitted using the GraphPad Prism software.

Since treatment of HepG2 cells with BRD0418 increases the levels of LDL receptor in cell lysates and decreases the levels of extracellular PCSK9 protein, we evaluated the effect of BRD0418 on cellular LDL uptake. We quantified the uptake of BODIPY-FL-conjugated human LDL at various time intervals in cells pretreated for 20 hours with BRD0418. Treatment with BRD0418 resulted in about 5-fold increase in the rate of labeled LDL uptake (Fig. 4A). In comparison, cholesterol depletion also resulted in about 5-fold increase in the rate of labeled LDL uptake, although the stimulation of uptake by BRD0418 in 10% serum was about 5-fold higher than stimulation achieved through cholesterol starvation in 0.5% lipid-deprived serum (Fig. 4B). BRD0418 produced the half-maximal effect on LDL uptake at 3.3 μM (Fig. 4C), which corresponded to its effects on *LDLR* and *PCSK9* transcript expression.

### *TRIB1* is upregulated by oncostatin M

**Fig 5:**
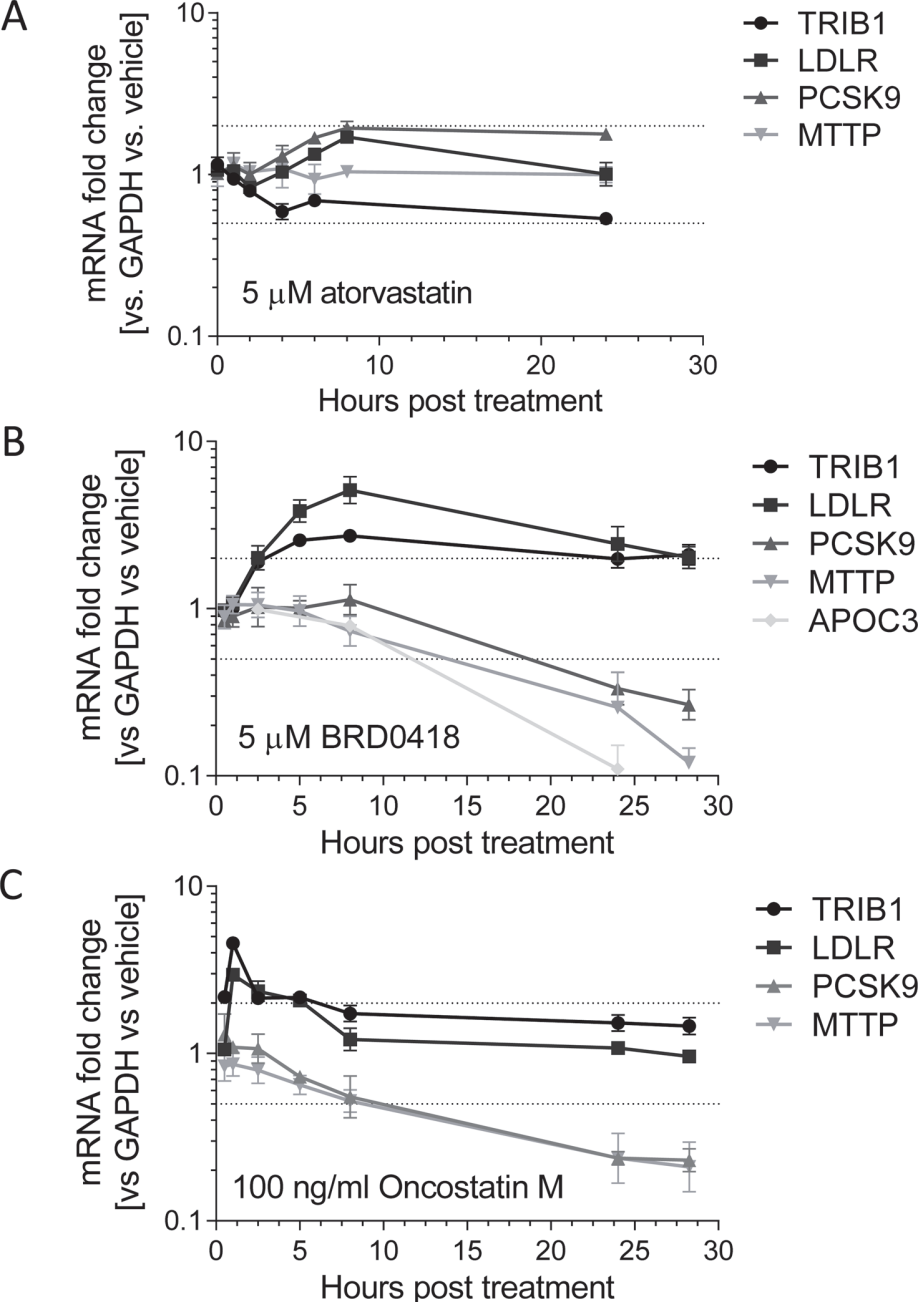
Kinetics of gene expression modulation by atorvastatin, BRD0418 and oncostatin M. HepG2 cells were treated with the indicated concentrations of stimulating agents and the changes in gene expression were measured at indicated time points by qRT-PCR. Fold change of expression (relative expression normalized to *GAPDH*, compared to vehicle control) was calculated for *TRIB1* (circle), *LDLR* (square), *PCSK9* (triangle), *MTTP* (inverted triangle) and *APOC3* (diamond).

Since, in HepG2 cells treated with BRD0418, the change in *LDLR* expression appears to be decoupled from changes in expression of *PCSK9* and cholesterol metabolic genes, we sought to more closely characterize the gene-expression changes in a time-course experiment. In contrast to atorvastatin, which induced a nearly 2-fold increase of *LDLR* and *PCSK9* mRNA levels and did not significantly affect expression of *TRIB1* (Fig. 5A), BRD0418 robustly upregulated *TRIB1* and *LDLR* expression but strongly reduced *PCSK9*, *MTTP* and *APOC3* expression (Fig. 5B). Maximal increase in *LDLR* and *TRIB1* levels occurred after 6–8 hours, whereas the decrease in *PCSK9*, *MTTP* and *APOC3* levels was a late response and occurred after 24 hours of treatment. We also tested the effects of oncostatin M (OSM), as it was previously demonstrated to upregulate *LDLR* and downregulate *PCSK9* expression in HepG2 cells [32]. Interestingly, OSM strongly induced the expression of *TRIB1*, and resulted in a pattern of changes resembling the responses to BRD0418 (Fig. 5C), except that the peak responses occurred after one hour rather than six hours.

### Effects of BRD0418 treatment and cholesterol depletion are additive

**Fig 6:**
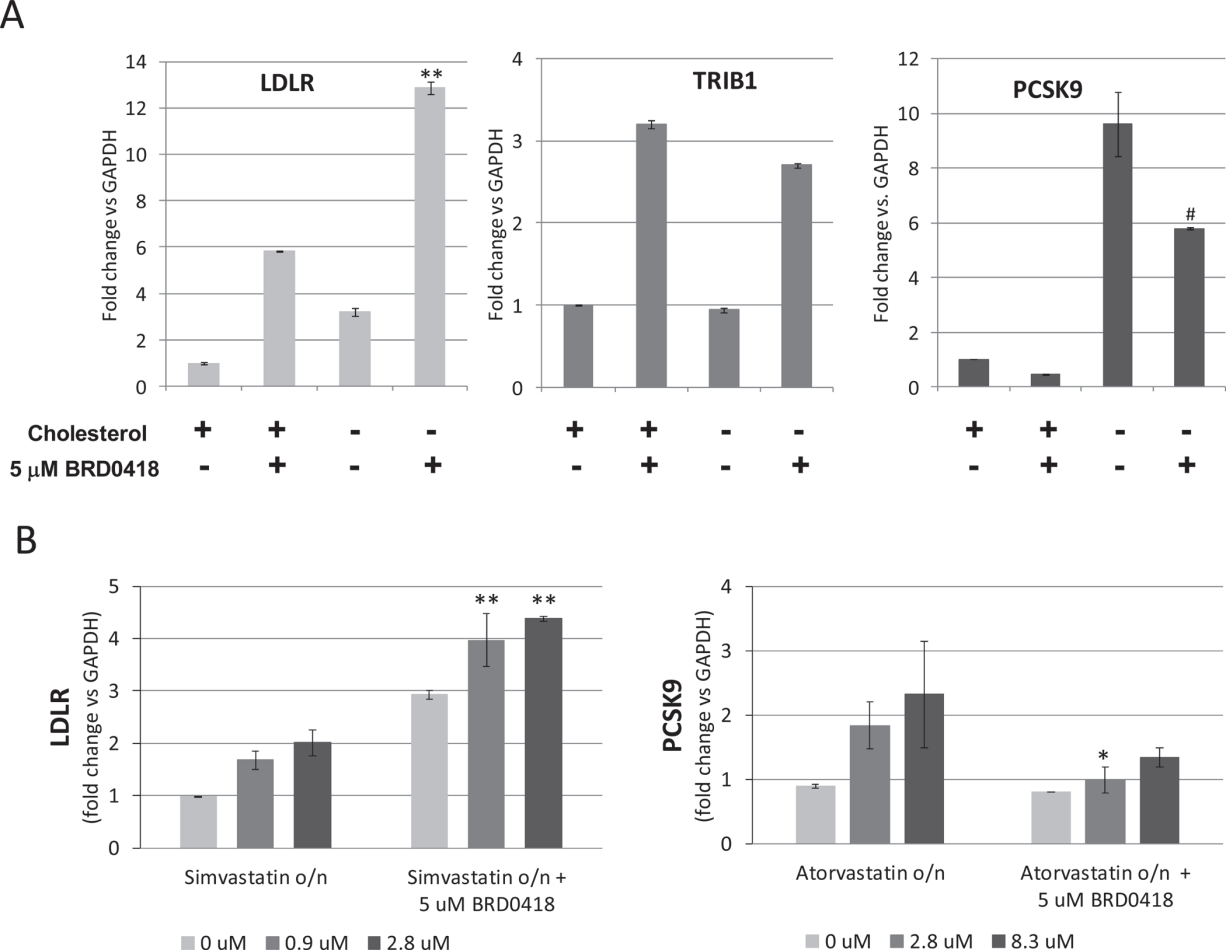
Effects of BRD0418 on *LDLR* and *PCSK9* expression are additive with the effects of cholesterol depletion and with the effects of statins. (A) HepG2 cells pre-grown for 24 hours in the lipoprotein-depleted media in the presence or absence of cholesterol were treated with 5 μM BRD0418 or with DMSO and the levels of transcripts were measured by qRT-PCR 6 hours (for *LDLR* and *TRIB1*) and 24 hours (for *PCSK9*) after compound treatment. Data represent mean ± S.D. (*error bars*) for two replicates. *P* values were determined with Student’s t-test for the comparison with the single stimulus induction. **, *p*<0.01; #, *p* = 0.15. (B) HepG2 cells pre-grown for 24 hours in the presence of indicated concentrations of a statin were treated for 6 hours (left panel) or for 24 hours (right panel) with 5 μM BRD0418, and the levels of *LDLR* (left panel) and *PCSK9* (right panel) transcripts were quantified by qRT-PCR. Data represent mean ± S.D. (*error bars*) for two replicates. P values were determined with Student’s t-test for the comparison with statin induction. *, *p*<0.05; **, *p*<0.01.

The decoupling of *LDLR* from *PCSK9* responses to BRD0418 treatment suggested an SREBP-2-independent mechanism of regulation. To verify this supposition, we tested the effects of BRD0418 on HepG2 cells under conditions of cholesterol depletion. Expectedly, after a 24-hour incubation in lipoprotein-depleted media, the levels of *LDLR* and *PCSK9* transcripts were increased 3- and 9-fold, respectively, compared to cholesterol-supplemented media (Fig. 6A). *TRIB1* levels were not regulated by cholesterol, and remained the same under low- and high-cholesterol growth conditions. Treatment of cholesterol starved cells with 5 μM BRD0418 elevated *LDLR* an additional 4-fold to reach a combined 12-fold increase, but decreased *PCSK9* transcript level by 40%. This result demonstrates that the effects of BRD0418 are additive with the effects of cholesterol depletion. We then sought to determine whether the effects of BRD0418 were additive with the effects of pharmacologically induced cholesterol depletion. Combination of overnight treatment with a statin with a subsequent treatment with BRD0418 had an additive effect on the induction of *LDLR* expression (Fig. 6B) and led to significantly reduced expression of *PCSK9* (Fig. 6B). These results further support the conclusion that BRD0418 acts through a SREBP-2-independent mechanism.

### Limited effect of BRD0418 on non-hepatic cells

*TRIB1 is* ubiquitously expressed in most tissues with high levels of expression found in lungs, lymphocytes, pancreas, liver and placenta [14]. To begin address tissue selectivity of *TRIB1* induction by BRD0418 we tested the levels of *TRIB1* and *LDLR* transcripts in twelve cell lines originating from various tissues (Table 3). Although we observed induction of *TRIB1* in a muscle and lung cell lines the strongest and the most consistent effects on both genes were found in cells of hepatic origin.

**Table 3:** Effect of BRD0418 on *TRIB1* and *LDLR* levels in cell lines of various origins. Twelve cell lines were subjected to treatment with BRD0418 in concentrations ranging from 0.2 to 25 μM and tested for *TRIB1* and *LDLR* up-regulation relative to *GAPDH* expression after 6 hour and 24 hour exposure to the compound. The values listed in the table represent the lowest concentration of the compound at which 2-fold up-regulation (or maximal up-regulation shown in brackets) of the measured transcript was observed. IA, inactive.

Cell Line	Tissue	6 hour		24 hour	
		*TRIB1*	*LDLR*	*TRIB1*	*LDLR*
HepG2	Liver	0.8	0.8	0.6	0.8
Huh7	Liver	0.8	0.7	0.8	0.8
JHH5	Liver	IA	3.0 [1.6]	IA^1^	IA^1^
CAPAN2	Pancreas	IA	IA	6.3	IA
DU145	Prostate	IA	IA	IA	IA
HCT8	Large Intestine	1.6	IA	IA	IA
HS683	Glioma	IA	6.3	IA	6.3
MFE296	Endometrium	IA	IA	3.0	IA
SKMES1	Lung	IA	6.3	0.4	IA
SNU216	Stomach	IA	IA	IA	IA
WM115	Skin	IA	IA	IA	IA
KYM1	Muscle	0.8	1.5 [1.6]	IA^1^	IA^1^
MDAMB361	Breast	IA	IA	IA	IA

^1^ Effect on GAPDH observed

### Effects of BRD0418 are MAPK-dependent

**Fig 7:**
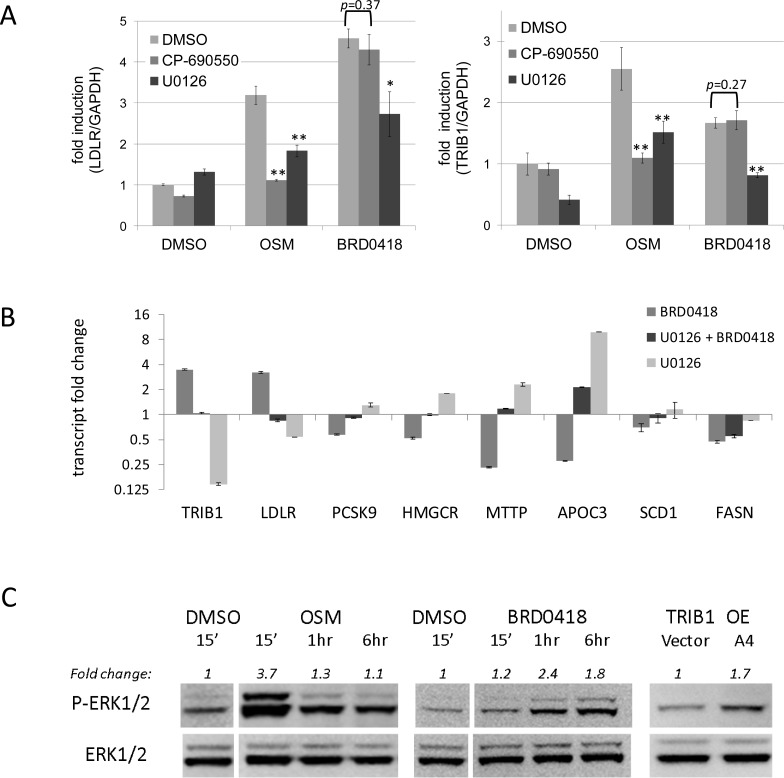
Early and late responses to BRD0418 depend on stimulation of MAPK^Erk^ signaling. (A) Levels of *LDLR* (left panel) and *TRIB1* (right panel) transcript measured by qRT-PCR in HepG2 cells pretreated for 1 hour with 1 μM JAK inhibitor CP-690550 and with 5 μM MEK inhibitor U0126 followed by 6 hour treatment with 10 μM BRD0418 or 1 hour treatment with 100 ng/mL oncostatin M. Data represent mean ± S.D. (*error bars*) for three replicates. P values were determined with Student’s t-test for the comparison with stimulated sample. *, *p*<0.05; **, *p*<0.01. (C) Levels of eight transcripts measured by qRT-PCR in HepG2 cells pretreated for 1 hour with 5 μM U0126 followed by 24 hour treatment with 10 μM BRD0418 or treated for 24 hours with individual drugs alone. Data represent mean fold change relative to vehicle treated cells ± S.D. (error bars) for three replicates. *P* values determined with the Student’s t-test for comparison of BRD0418 treated samples ± U0126 were less than 0.01 for all transcripts except for *SCD1* and *FASN*. (C) Stimulation of ERK phosphorylation by treatment of HepG2 cells with BRD0418 and by overexpression of *TRIB1* gene. Cells were incubated for 18 h in serum free minimal essential medium and treated with 100 ng/mL OSM and 5 μM BRD0418 for the indicated time intervals and the total and phosphorylated ERK1/2 were detected by Western blot. HepG2 clones stably transfected with vector alone and with *TRIB1* plasmid (clone A4) were also incubated overnight in the serum free media prior to Western blot analysis. A representative image for three independent experiments is shown. The average stimulation of ERK1/2 phosphorylation in three experiments was 2.0±0.7-fold at 1 hour and 1.8±0.2-fold at 6 hours (mean ± S.D.). Fold change values represent relative intensity of phospho-ERK bands in comparison to DMSO controls for matching time points. The values were normalized for loading based on total ERK signal intensity.

The primary screening results and the subsequent L1000 analysis indicated that the responses of HepG2 cells to BRD0418 are in many aspects opposite to the responses to MEK1/2 inhibitors, suggesting possible involvement of the MAP kinase signaling pathway in mediating BRD0418 activity. To begin mapping the interaction of BRD0148 with signal transduction pathways we used two highly specific kinase inhibitors, CP-69550, a JAK inhibitor, which blocks receptor proximal signaling and U0126, a MEK inhibitor, which blocks MAPK^Erk^ signaling cascade. First, we evaluated effects of CP-690550 and U0126 on the stimulating activities of BRD0418 and OSM after short duration of treatment. As reported previously, the *LDLR* upregulating activity of OSM was both JAK- and MEK-dependent, whereas activity of BRD0418 was inhibited by the MEK inhibitor, but not by the JAK inhibitor (Fig. 7A left panel). Upregulation of *TRIB1* expression showed similar sensitivity to U0126 (Fig. 7A right panel). In addition, the basal level of *TRIB1* in HepG2 cells was reduced by U0126 treatment indicating tight regulation of *TRIB1* expression by MAPK^Erk^ signaling. We also examined the effects of MAPK^Erk^ blockade on late responses after 24h treatment with BRD0418. Similar as in case of stimulation of *TRIB1* and *LDLR* the downregulation of *PCSK9*, *MTTP*, *APOC3* and *HMGCR* transcripts was also reversed in the presence of U0126 pretreatment (Fig. 7B). Notably, most of the genes tested—with the exception of *SCD1* and *FASN*, which were not strongly affected by U0126—showed opposite responses to treatments with individual drugs confirming the results of L1000 profiling.

Next we found that BRD0418, similar to OSM, stimulates signaling through MAPK^Erk^, as treatment of HepG2 cells with those agents led to ERK1/2 phosphorylation, which temporally preceded upregulation of *TRIB1* expression (Fig. 7C). The response to OSM was rapid and short-lived, as it was strong at 15 minutes and reverted to basal level 1 hour after the treatment, whereas the response to BRD0418 occurred 1 hour after treatment and persisted longer, remaining strong at 6 hours after the treatment. Interestingly, constitutively elevated expression of *TRIB1* in HepG2 cells stably transfected with the expression plasmid also resulted in elevated phosphorylation of ERK (Fig. 7C). These data indicate that BRD0418 affects elements of the network that operate downstream of JAK and upstream of the ERK signaling pathway, leading to broad MEK1/2-dependent transcriptional changes. It is unlikely that BRD0418 acts directly through nuclear hormone receptors known to modulate lipid metabolism, as PPARγ, PPARα, FXR and RAR agonists had no effect on *TRIB1* transcript levels in HepG2 cells (data not shown).

### Effects of genetic perturbation of *TRIB1* expression on lipid metabolic genes and on BRD0418 responses in HepG2 cells

**Fig 8:**
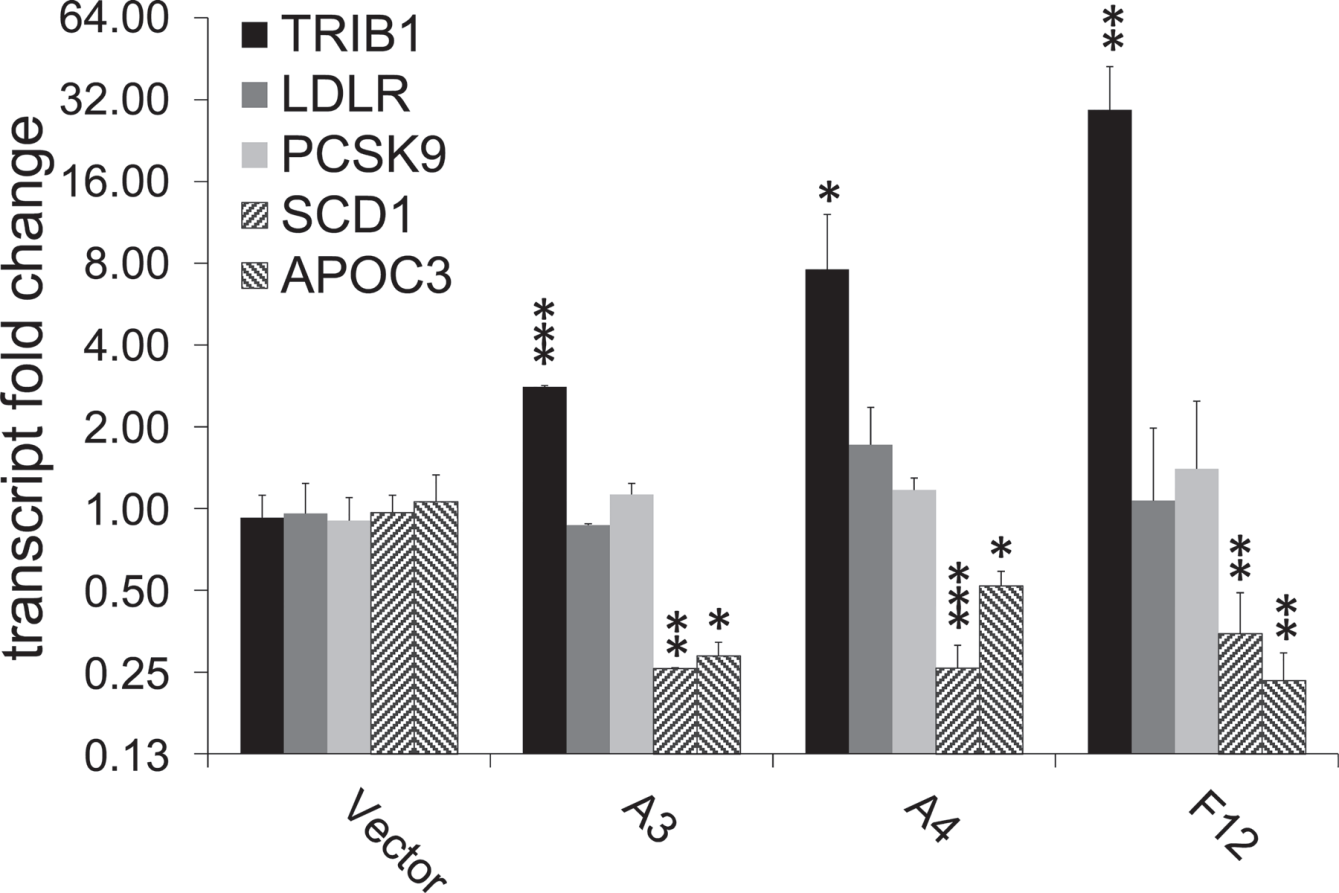
Effect of *TRIB1* overexpression on transcript levels of selected lipoprotein metabolic genes. Transcript levels of *LDLR*, *PCSK9*, *SCD1* and *APOC3* were measured by qRT-PCR in clones of HepG2 cells transfected with a plasmid carrying *TRIB1* gene under control of the CMV promoter. Data represent means **±** S.D. (*error bars*) for three replicates. *, *p*<0.05; **, *p*<0.01; ***, *p*<0.001 as determined with Student’s t-test for the comparison with vector control.

Changes in the level of *TRIB1* expression in animal models have been reported to regulate expression of TG but not cholesterol biosynthetic genes [13, 14]. To evaluate the role of *TRIB1* in lipid metabolism in HepG2 cells and in mechanism of BRD0418 action we constructed HepG2 clones stably overexpressing or lacking the expression of *TRIB1*. First, to help determine whether changes in *LDLR* and *PCSK9* expression entailed by BRD0418 are related to upregulation of *TRIB1* expression or are independently induced by BRD0418 treatment, we measured the level of all three transcripts in a collection of clones of HepG2 cells selected after transfection with *TRIB1* expression vector. We found no changes in the levels of *LDLR* or *PCSK9* transcripts in clones in which *TRIB1* overexpression ranged between 3-fold and 30-fold (Fig. 8). In contrast, the expression levels of *SCD1* and *APOC3* were significantly decreased in all clones (Fig. 8). These results indicate that the regulation of LDL uptake genes by BRD0418, at least under conditions of chronic overexpression of *TRIB1*, is not affected by *TRIB1* upregulation.

**Fig 9:**
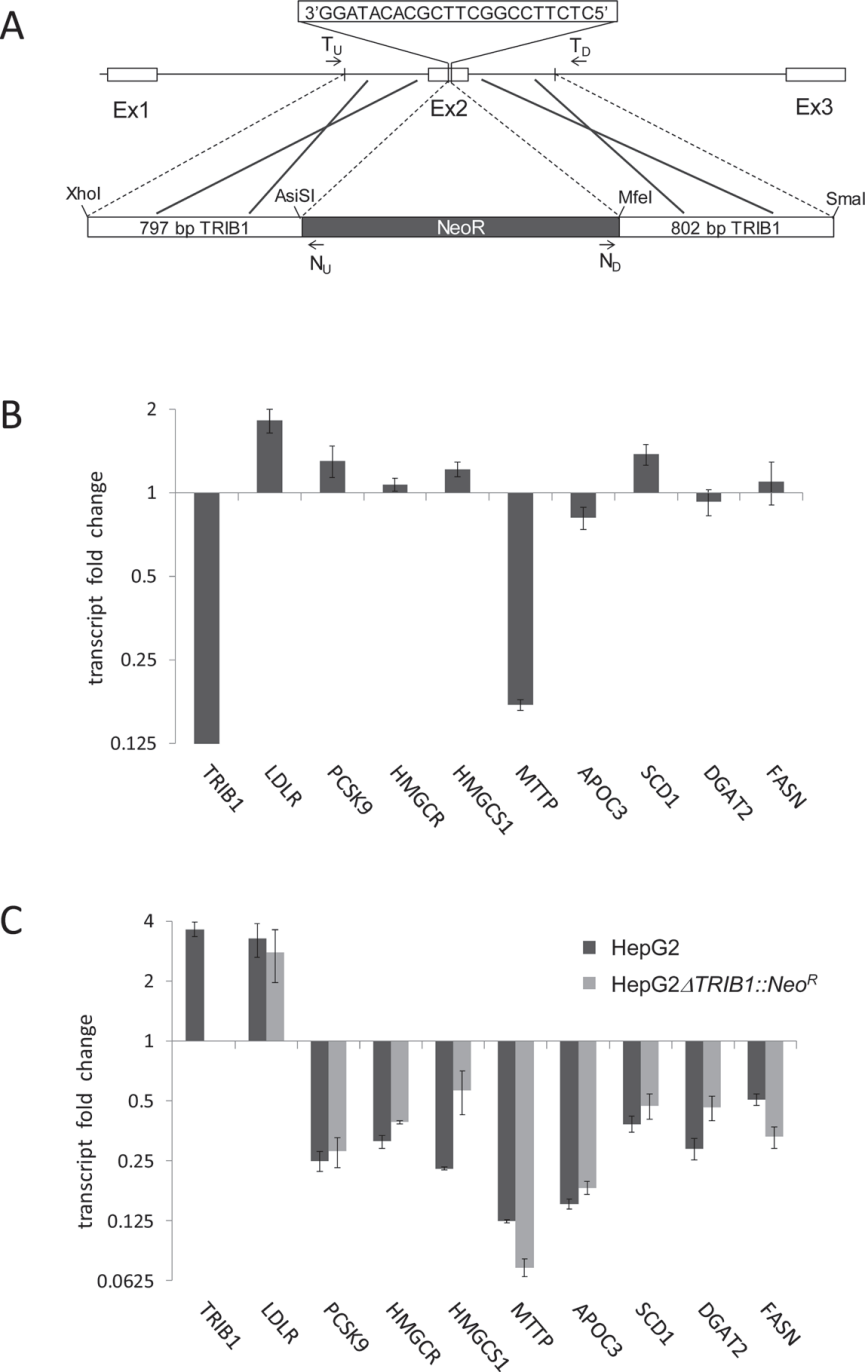
Effects of BRD0418 on expression of selected marker genes in HepG2*ΔTRIB1*::*Neo* ^*R*^ cell line carrying chromosomal deletion/disruption of *TRIB1* locus. (A) Schematic representation of CRISPR-Cas9 induced inactivation of *TRIB1* gene in HepG2 cells. The indicated 22 bp target sequence in exon 2 of *TRIB1* gene was used to design Cas9 guide RNA. The replacement of the target sequence in the chromosome with the Neo^R^ gene was induced by co-transfecting cells with the CRISPR-Cas9 plasmid together with the homologous recombination repair template consisting of Neo^R^ gene cassette and two ∼800 bp fragments from the *TRIB1* locus that flank the target sequence. Location of PCR primers used for identifying the Geneticin resistant clone that carries the designed chromosomal rearrangement is indicated by arrows. (B) Changes in the steady state transcript levels in HepG2*ΔTRIB1*::*Neo* ^*R*^ cells comparing to parental HepG2 cells. The relative expression level of indicated lipid metabolic genes normalized to expression level of *GAPDH1* was measured by qRT-PCR 48 hours after plating cells in 384-well plate. The mean values for six replicates ± S.D. (*error bars*) are shown for the representative of three independent experiments. (C) Changes in transcript levels in HepG2 and in HepG2*ΔTRIB1*::*Neo* ^*R*^ cells in response to 24 h treatment with 10 μM BRD0418. The values are normalized to DMSO controls for each strain and represent mean fold change for six replicates ± S.D. (*error bars*).

Second, given the established effects of *TRIB1* overexpression on lipogenic genes we examined the impact of *TRIB1* knockout in HepG2 cells on modulation of lipid gene expression by BRD0418. Since the *TRIB1* mRNA has a very high turnover rate [33] and our attempts to downregulate *TRIB1* expression with 14 different shRNA constructs did not yield more than a 50% reduction in the transcript level, we constructed *TRIB1* knockout HepG2 cell line using a CRISPR-Cas9 gene editing approach [34]. HepG2 cells harbor an inactivating mutation in the *PRKDC* gene that is essential for non-homologous end joining (NHEJ) mechanism of DNA repair [35]. This information, retrieved from the CCLE database (http://www.broadinstitute.org/ccle/home; 1 Dec 2014), provided plausible explanation for the resistance of this cell line to NHEJ based gene editing procedures that we encountered in early attempts. However, induction of the homologous recombination (HR)-based DNA repair by co-transfecting HepG2 cells with CRISPR-Cas9 plasmid together with the HR repair template DNA (Fig. 9A) yielded from 171 Geneticin resistant clones, 17 heterozygous clones carrying one copy of *ΔTRIB1*::*Neo* ^*R*^ allele and one clone with homozygous deletion of the wild-type *TRIB1* locus that carried *ΔTRIB1*::*Neo* ^*R*^ allele in both copies of chromosome 8. PCR amplification of genomic DNA from this cell line produced the expected size DNA fragments for T_U_/N_U_, N_D_/T_D_ and T_U_/T_D_ primer pairs (1167 bp, 1228 bp and 3395 bp, respectively (Fig. 9A)) but failed to produce the 2020 bp intact *TRIB1* fragment present in reactions carried out for the parental cell line or for heterozygous clones. Moreover, qRT-PCR reactions carried out for the HepG2 *ΔTRIB1*::*Neo* ^*R*^ cell line using the Taqman primers flanking the target sequence (Table 1) failed to amplify any products or resulted in ΔCt values at least 10 cycles higher than for the HepG2 cells consistent with complete abrogation of *TRIB1* expression in those cells.

Next we compared levels of expression of key lipid metabolic genes in HepG2 cells and HepG2 *ΔTRIB1*::*Neo* ^*R*^ cells and compared responses of both cell lines to treatment with 10 μM BRD0418. Abrogation of *TRIB1* expression in HepG2 cells resulted in a greater than 5-fold reduction in *MTTP* expression, a 2-fold upregulation of *LDLR* expression and a smaller but consistent elevation of *PCSK9* and *SCD1* transcript levels (Fig. 9B) further confirming the modulatory function of *TRIB1* in hepatic lipid metabolism. Asymmetrically to the *TRIB1* overexpression results, the *TRIB1* knockout had no significant effect on BRD0418 driven downregulation of *SCD1* and *APOC3*, genes involved in TG synthesis and lipoprotein assembly (Fig. 9C), indicating that BRD0418 effects are *TRIB1* independent or that in the absence of *TRIB1* expression, BRD0418 treatment can evoke a compensatory response. Therefore, *TRIB1* overexpression is sufficient but not necessary to induce downregulation of *SCD1* and *APOC3* expression in HepG2 cells. As expected from *TRIB1* overexpression results, the *TRIB1* knockout had no large effect on responses of cholesterol metabolic genes to BRD0418 treatment (Fig. 9C).

## Discussion

The *TRIB1* locus has been linked through GWAS to changes in blood lipid levels and to risk of CAD and MI. Genetic studies in mice revealed that increased expression of *Trib1* in liver is associated with decreased apoB-100 secretion and VLDL production and decreased plasma TG and cholesterol levels. In the present study, we identified, through a high-throughput qRT-PCR screen, small-molecule upregulators of *TRIB1* expression and characterized the effects of the lead compound, BRD0418, on lipid metabolism in HepG2 hepatoma cells. BRD0418 increased expression of *TRIB1* 4-fold in HepG2 cells in a dose-dependent manner (EC_50_ = 2.5 μM). Accordingly, BRD0418 also decreased the rate of TG synthesis and inhibited secretion of apoB-100. Unexpectedly, however, treatment with BRD0418 also led to a 4-fold upregulation of *LDLR* expression, a 3-fold increase of the LDL receptor protein and a proportional increase in the rate of LDL uptake. Furthermore, BRD0418 was equally potent in inhibiting *PCSK9* mRNA expression and protein secretion, inhibiting PCSK9 protein secretion by 70% after 24-hour treatment.

The effects of BRD0418 on *LDLR* and *PCSK9* expression appeared to be independent of *TRIB1* upregulation, as overexpression of *TRIB1* cDNA had no impact on *LDLR* and *PCSK9* transcript levels. As expected, based on previous studies in mice [14], plasmid-driven *TRIB1* overexpression led to downregulation of the expression levels of *SCD1*, a rate-limiting gene in hepatic TG synthesis. Interestingly, we found equally strong correlation between overexpression of *TRIB1* cDNA in HepG2 cells and downregulation of *APOC3* expression. ApoC-III is a major component of lipoproteins linked to the rate of VLDL secretion and a negative regulator of lipoprotein lipase and blood triglycerides [36, 37]. *APOC3* is an independent CHD and MI risk factor, and hypomorphic apoC-III variants are cardioprotective [38–40]. Treatment of HepG2 cells with BRD0418 also downregulated, as a part of late response, *APOC3* expression. We detected significant changes in steady state levels of genes involved in VLDL production (*MTTP*) and LDL uptake (*LDLR* and *PCSK9*) in HepG2 *ΔTRIB1*::*Neo* ^*R*^ cells that lack *TRIB1* expression due to CRISPR-Cas9 induced inactivation of the chromosomal *TRIB1* locus. This observation further highlights the role of *TRIB1* in regulation of hepatic lipoprotein metabolism albeit the observed changes differ from those reported for mice, where *Trib1* knockout was associated with elevated expression of *Fasn* and *Scd1* and no changes in *Mttp* expression in liver [14]. HepG2 *ΔTRIB1*::*Neo* ^*R*^ cells responded to BRD0418 treatment with similar magnitude and pattern of responses as parental HepG2 cells (Fig. 9C) indicating that changes in *SCD1* and *APOC3* expression produced by BR0418 are also independent of *TRIB1* upregulation. Alternatively, it is likely that TRIB1 plays a role in driving some of the responses to the drug but its absence in the knockout strain allows for engagement of a compensatory mechanism (e.g. through activity of TRIB2 or TRIB3, which is also upregulated in response to BRD0418 treatment (Fig. 3)) that masks effects of the *TRIB1* knockout.

L1000 transcriptional profiling indicated that BRD0418 had a broad effect on lipid metabolic genes, and comparison of L1000 profiles revealed that cells treated with MEK inhibitors U0126 and PD-98059 produced opposite responses compared to cells treated with the BRD0418 and its analogs. Upon further characterization, we found that the responses to BRD0418 are MEK-dependent, as they are blocked by treatment with U0126, and that BRD0418 stimulates ERK1/2 phosphorylation. MAPK^Erk^ signaling has been previously shown to regulate apoB secretion in HepG2 cells, as ERK inhibition stimulates VLDL assembly and secretion [41, 42], whereas active ERK signaling is required for downregulation of apoB secretion by nobiletin [43]. ERK signaling has also been shown to be required for downregulation of *PCSK9* expression by OSM [32] and for stimulation of *LDLR* expression by berberine [44]. Activation of ERK1/2 signaling by BRD0418 was reflected in the L1000 transcriptional profile through induction of early-response genes including *EGR1* (Fig. 3). EGR1 has been previously reported to drive upregulation of *LDLR* expression in response to OSM [45], which is known to stimulate LDL uptake both *in vitro* and *in vivo* [46, 47]. Other treatments that stimulate ERK phosphorylation in hepatoma cells that had been previously shown to upregulate LDL uptake include HGF [48], FGF21 [49], hGH [50], PMA [51] and AICAR [52]. The AICAR effect was shown to stem from stimulation of ERK signaling, rather than from activation of AMPK [52].

Interestingly, TRIB1 itself has been shown to directly interact with MEK1 and to enhance ERK signaling [53, 54]. A novel connection between mitochondrial function, ERK1/2 signaling and *TRIB1* expression was recently established in a report demonstrating the MEK-dependent upregulation of *TRIB1* expression by the oxidative phosphorylation inhibitor Oligomycin A [31]. Treatment of HepG2 cells with BRD0418 had no obvious effects on mitochondrial function, as measured by cellular ATP levels in cells grown on galactose, or on mitochondrial membrane depolarization, as measured by JC-1 staining (data not shown). However, we cannot preclude more nuanced effects of BRD0418 on mitochondria as we observed that berberine, which was reported to inhibit mitochondrial complex I [55], and AICAR, which simulates changes in cellular AMP/ATP ratio, also strongly stimulated upregulation of *TRIB1* and *LDR1* expression in HepG2 cells (S1 Fig. AICAR and berberine treatment). Effects of AICAR were observed at relatively high concentrations of the compound (*TRIB1* EC_50_ = 980 μM) consistent with reported doses required to stimulate *LDLR* expression in HepG2 cells [52] and with doses needed to produce physiological responses [56]. Our observation differs from a recent report indicating that AICAR has no effect on *TRIB1* expression in HepG2 cells perhaps due to a difference in a tested drug dose [31].

The differential effect of BRD0418 on genes regulating LDL uptake (increased expression of *LDLR* and decreased expression of *PCSK9*) was preserved in cells grown under conditions of cholesterol depletion, and was additive with effects of statins, suggesting that BRD0418 is not acting along the SREBP2 regulatory pathway. The decoupled response of *LDLR* and *PCSK9* expression in HepG2 cells treated with BRD0418 resembled the responses to OSM, a cytokine involved in liver development and regeneration [32], and to berberine, a plant alkaloid with pleiotropic effects and undefined mode of action [57]. OSM was efficacious in lowering triglyceride and cholesterol levels in hyperlipidemic syrian hamsters demonstrating that the OSM signaling pathway that drives changes in lipoprotein metabolism in hepatoma cells is also operational *in vivo* [47]. Berberine was also shown to have hypolipidemic activity, both in animal models and in patients [44], however the efforts to optimize its PK properties and to identify its molecular target(s) are hampered by the nature of its chemical scaffold.

The molecular target of BRD0418 is currently unknown, but our data indicate that its perturbation produces a broad response that involves activation of MAPK^erk^ signaling followed by upregulation of a number of early response genes including *TRIB1* and *LDLR* and leads to remarkable change in the lipoprotein metabolism, which results in a decrease in VLDL production and upregulation of LDL uptake in cells of hepatic origin. Identification of the molecular target of BRD0418 should help to unravel how those two arms of hepatic lipoprotein metabolism are coordinated. Overexpression of *TRIB1* has been also linked to leukemia [18] and, while BRD0418 has limited effects on non-hepatic cells and the potency of *TRIB1* as an oncogene has not yet been fully evaluated, the oncogenic potential of *TRIB1* inducing agents will need to be closely monitored in future animal studies.

BRD0418 was identified from a library designed using the diversity oriented synthesis (DOS) strategy, which yields molecules with diverse stereochemistries and skeletons and provides modular synthetic pathways to facilitate downstream medicinal chemistry efforts. Initial SAR derived from the closer characterization of the screening hits from the benzofuran library indicated that the R^1^ position of the benzofuran scaffold can tolerate a wider range of substituents than R^2^ position, and suggests possible derivatization strategies for BRD0418 to generate affinity and cross-linking probes that can be employed for target-identification efforts.

Collectively, our data highlight the presence in hepatic cells of a naturally occurring, MAPK^Erk^-dependent lipoprotein rheostat that through the two arms of lipoprotein metabolism can downregulate the VLDL output by decreasing TG synthesis and upregulate the LDL uptake by increasing the expression of LDL receptor on the cell surface. This rheostat can be naturally regulated by endogenous modulators like OSM or growth factors but can also be accessed by small-molecule natural products like berberine or by more chemically tractable synthetic compounds like BRD0418 described here. Identification and targeting of this new regulatory node will help to find new hypolipidemic agents and to evaluate their therapeutic utility.

## Supporting Information

S1 Dataset L1000 data. (XLSX)

S1 Fig AICAR and berberine treatment. Changes in transcript levels in response to serially diluted doses of BRD0418, berberine and AICAR were measured in HepG2 cells by qRT-PCR 6 hours post treatment. Data represent mean fold change ± S.E (error bars) of three replicates.
(TIF)

S1 Protocol Synthesis of BRD0418. (DOCX)

S1 Table GO enrichment analysis. GO terms (process) enriched in the list of L1000 genes affected by the treatment of HepG2 cells with 10 μM BRD0418 were identified using the web tool GOrilla (cbl-gorilla.cs.technion.ac.il/). Only terms with FDR < 1.0E-1 and P-value < 1.0E-4 are listed in the table. Since L1000 approach provides an abbreviated transcriptional profile comprising 961 genes only one term in this analysis reached a significance level of FDR < 0.05.
(DOCX)
