# Supplementary figures and images for: Modulators of Hepatic Lipoprotein Metabolism Identified in a Search for Small-Molecule Inducers of Tribbles Pseudokinase 1 Expression
Source: PLoS One. 2015 Mar 26;10(3):e0120295. doi: 10.1371/journal.pone.0120295 (PMC4374785; doi:10.1371/journal.pone.0120295)

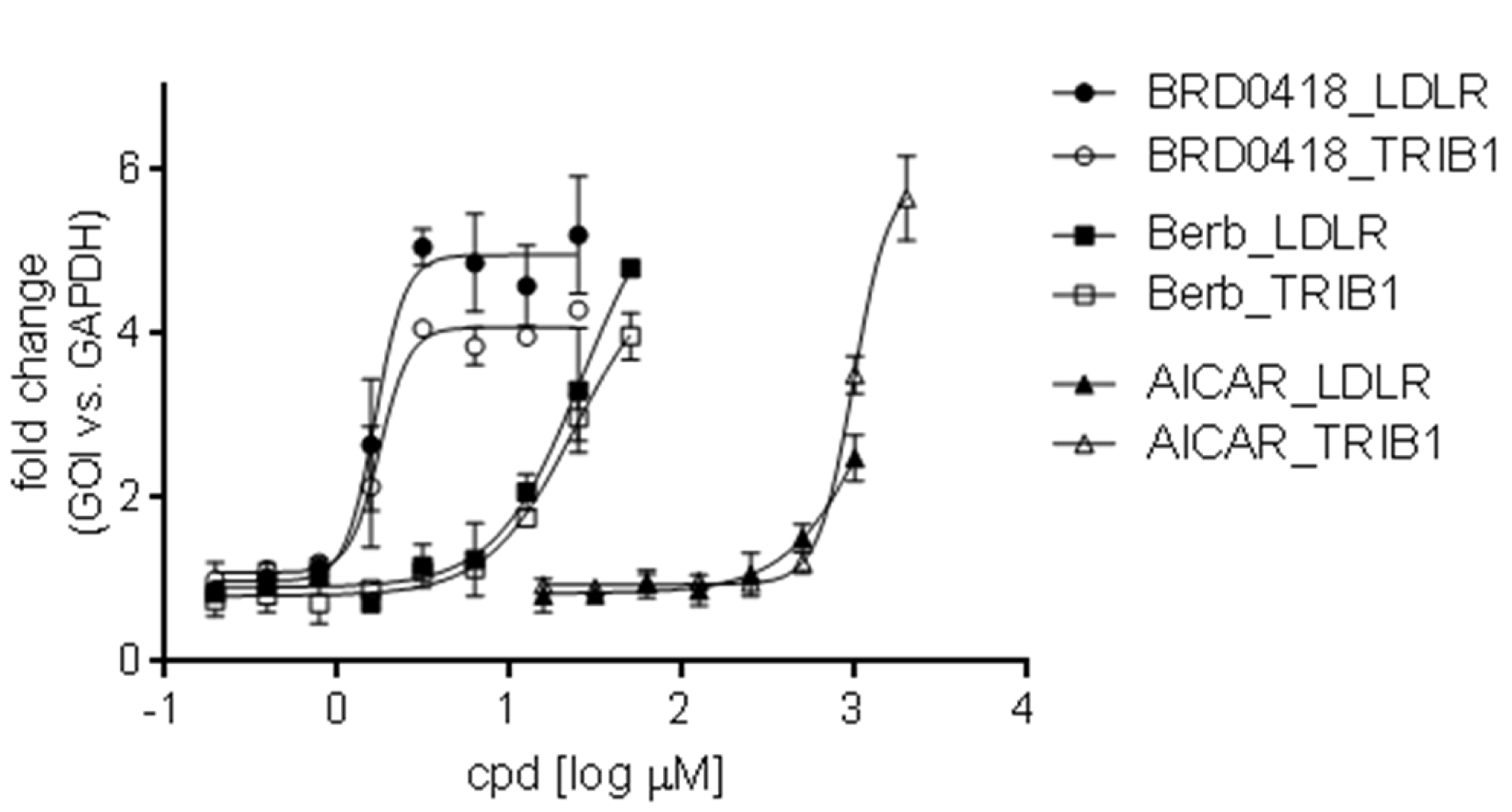

Supplement: S1 Fig — Changes in transcript levels in response to serially diluted doses of BRD0418, berberine and AICAR were measured in HepG2 cells by qRT-PCR 6 hours post treatment. Data represent mean fold change ± S.E (error bars) of three replicates. (TIF) [file pone.0120295.s002.tif]
