# Supplementary material for: Modulators of Hepatic Lipoprotein Metabolism Identified in a Search for Small-Molecule Inducers of Tribbles Pseudokinase 1 Expression
Source: PLoS One. 2015 Mar 26;10(3):e0120295. doi: 10.1371/journal.pone.0120295 (PMC4374785; doi:10.1371/journal.pone.0120295)
Supplement: S1 Protocol — (DOCX) [file pone.0120295.s003.docx]

**S1_Protocol.** *Synthesis of BRD0418.* General Methods**.** All oxygen and/or moisture-sensitive reactions were carried out under N_2_ atmosphere in glassware that had been flame-dried under a vacuum (~0.5 mmHg) and purged with N_2_ prior to use. All reagents and solvents were purchased from commercial vendors and used as received or synthesized according to the footnoted references. ^1^H and ^13^C NMR spectra were recorded on 300 and/or 500 MHz spectrometers. All chemical shifts are reported in parts per million (δ) referenced to residual non deuterated solvent. Data are reported as follows: chemical shifts, multiplicity (br = broad, s = singlet, d = doublet, t = triplet, q = quartet, p = pentet, m = multiplet; coupling constant(s) in Hz; integration). Unless otherwise indicated, NMR data were collected at 25 °C. Flash chromatography was performed using 40−60 μm silica gel (60 Å mesh) with the indicated solvent. Analytical thin layer chromatography (TLC) was performed on 0.25 mm silica gel 60-F plates. Visualization was accomplished with UV light and aqueous potassium permanganate or ceric ammonium molybdate stain followed by heating. High resolution mass spectra were obtained using a LC-MS coupled with a quadrupole.

**2-((1S,3S,4aS,9aR)-6-(dimethylamino)-1-(hydroxymethyl)-3,4,4a,9a-tetrahydro-1H-pyrano[3,4-b]benzofuran-3-yl)-N-(4-phenoxybenzyl)acetamide (BRD0418).** To a solution of methyl 2-((1S,3S,4aS,9aR)-6-amino-1-(((tert-butyldiphenylsilyl)oxy)methyl)-3,4,4a,9a-tetrahydro-1H-pyrano[3,4-b]benzofuran-3-yl)acetate (Compound ***ent*-10a** from ref. [1]) (135.0 mg, 0.254 mmol, 1.0 equiv) in DCE (2.5 mL) was added formaldehyde (37% wt. in water, 94.0 mL, 1.270 mmol, 5.0 equiv). The reaction mixture was stirred at rt for 30 min, then sodium triacetoxyborohydride (270.0 mg, 1.270 mmol, 5.0 equiv) was added, and stirred at rt overnight. The reaction mixture was diluted with DCM (5 mL) and a saturated solution of NaHCO_3_ (6 mL) was added. The mixture was extracted with DCM (x3), and the combined organic layers were dried over Na_2_SO_4_. After filtration, excess solvent was removed in vacuo to afford a crude residue, which was purified by chromatography on silica gel (gradient: 0-60% EtOAc in hexanes) to provide 97.0 mg (68%) of the dimethyl aniline as a colorless oil.

To a solution of the resulting oil (97.0 mg, 0.173 mmol, 1.0 equiv) in THF/MeOH/H_2_O (5/2/2, 1.70 mL) was added potassium trimethylsilanolate (33.0 mg, 0.260 mmol, 1.5 equiv). The reaction mixture was stirred at rt for 24 hours. The reaction was then quenched with a saturated solution of aqueous ammonium chloride, and the reaction mixture was extracted with EtOAc (x3). The combined organic layers were washed with brine and dried over Na_2_SO_4_. After filtration, excess solvent was removed in vacuo to provide 94.0 mg (99%) of the carboxylic acid, which was used for next step without further purification.

To a solution of the carboxylic acid (94.0 mg, 0.173 mmol, 1 equiv) in DCM (1.73 mL) were added Hunig’s base (89.0 mL, 0.519 mmol, 3.0 equiv) and (4-phenoxyphenyl)methanamine (47.0 mL, 0.260 mmol, 1.5 equiv), followed by PyBOP (126.0 mg, 0.242 mmol, 1.4 equiv). The reaction mixture was stirred at rt for 2 hours. The reaction was then quenched with water, and the reaction mixture was extracted with DCM (x3). The combined organic layers were washed with brine and dried over Na_2_SO_4_. After filtration, excess solvent was removed in vacuo to afford a crude residue, which was purified by chromatography on silica gel (gradient: 0-10% MeOH in DCM) to provide 115.0 mg (91%) of the corresponding amide as a colorless oil.

To a solution of the resulting oil (115.0 mg, 0.158 mmol, 1.0 equiv) in THF (1.60 mL) was added pyridine hydrofluoride (70% HF, 79.0 mL, 0.633 mmol, 4.0 equiv). The reaction mixture was stirred at rt for 4 hours. The reaction was then quenched with methoxytrimethylsilane (869.0 mL, 6.33 mmol, 40.0 equiv) and stirred at rt for 40 min. The solvent was removed in vacuo to afford a crude residue, which was purified by chromatography on silica gel (gradient: 0-10% MeOH in EtOAc) to provide 55.0 mg (71%) of **BRD0418** as a white powder. ^1^H NMR (300 MHz, CDCl_3_): δ 7.35-7.31 (m, 2H), 7.21 (d, *J* = 6.0 Hz, 2H), 7.09 (dd, *J* = 9.0, 6.0 Hz, 1H), 6.99-6.92 (m, 3H), 6.80 (dd, *J* = 6.0, 3.0 Hz, 1H), 6.69 (d, *J* = 9.0 Hz, 1H), 6.61-6.56 (m, 2H), 4.51 (dd, *J* = 9.0, 9.0 Hz, 1H), 4.36 (d, *J* = 3.0 Hz, 2H), 3.87-3.73 (m, 3H), 3.65 (dd, *J* = 12.0, 6.0 Hz, 1H), 3.35-3.29 (br m, 1H), 2.88 (s, 6H), 2.46-2.43 (m, 2H), 2.23 (d, *J* = 12.0 Hz, 1H), 2.00-1.88 (m, 1H). ^13^C NMR (75 MHz, CDCl_3_) δ 170.43, 157.16, 156.54, 151.46, 146.95, 133.25, 129.76, 129.17, 128.66, 123.29, 119.04, 118.79, 113.66, 110.45, 109.47, 70.87, 63.38, 42.89, 42.65, 42.10, 39.51, 30.50. HRMS (ESI) calcd for C_29_H_33_N_2_O_5_ [M+H]^+^: 489.2389. Found: 489.239

1. Gerard B, Lee MD 4th, Dandapani S, Duvall JR, Fitzgerald ME, Kesavan S, et al. Synthesis of stereochemically and skeletally diverse fused ring systems from functionalized C-glycosides. The Journal of organic chemistry. 2013;78(11):5160-71. doi: 10.1021/jo4000916. PubMed PMID: 23692141; PubMed Central PMCID: PMC3752688.
