# Supplementary material for: Modulators of Hepatic Lipoprotein Metabolism Identified in a Search for Small-Molecule Inducers of Tribbles Pseudokinase 1 Expression
Source: PLoS One. 2015 Mar 26;10(3):e0120295. doi: 10.1371/journal.pone.0120295 (PMC4374785; doi:10.1371/journal.pone.0120295)
Supplement: S1 Table — GO terms (process) enriched in the list of L1000 genes affected by the treatment of HepG2 cells with 10 μM BRD0418 were identified using the web tool GOrilla (cbl-gorilla.cs.technion.ac.il/). Only terms with FDR < 1.0E-1 and P-value < 1.0E-4 are listed in the table. Since L1000 approach provides an abbreviated transcriptional profile comprising 961 genes only one term in this analysis reached a significance level of FDR < 0.05. (DOCX) [file pone.0120295.s004.docx]

**S1 Table.** GO terms enrichment analysis.

GO terms (process) enriched in the list of L1000 genes affected by the treatment of HepG2 cells with 10 μM BRD0418 were identified using the web tool GOrilla (cbl-gorilla.cs.technion.ac.il/). Only terms with FDR < 1.0E-1 and P-value < 1.0E-4 are listed in the table. Since L1000 approach provides an abbreviated transcriptional profile comprising 961 genes only one term in this analysis reached a significance level of FDR < 0.05.

| **GO term** | **Description** | **P-value** | **FDR q-value** | **Enrichment (N, B, n, b)** |
| --- | --- | --- | --- | --- |
| *24 h treatment. Downregulated genes.* | | | | |
| GO:0008152 | metabolic process | 6.13E-6 | 4.2E-2 | 1.03 (961,701,896,673) |
| GO:0071704 | organic substance metabolic process | 2.29E-5 | 7.85E-2 | 1.04 (961,665,867,622) |
| GO:0044238 | primary metabolic process | 2.91E-5 | 6.64E-2 | 1.04 (961,645,867,604) |
| GO:0071103 | DNA conformation change | 3.68E-5 | 6.3E-2 | 3.18 (961,19,223,14) |
| GO:0044265 | cellular macromolecule catabolic process | 4.03E-5 | 5.52E-2 | 1.96 (961,77,242,38) |
| GO:0044257 | cellular protein catabolic process | 5.25E-5 | 6E-2 | 30.03 (961,3,32,3) |
| GO:0044774 | mitotic DNA integrity checkpoint | 5.59E-5 | 5.47E-2 | 4.42 (961,11,178,9) |
| GO:0006695 | cholesterol biosynthetic process | 7.48E-5 | 6.41E-2 | 11.07 (961,7,62,5) |
| *24 h treatment. Upregulated genes.* | | | | |
| GO:0010033 | response to organic substance | 3.39E-5 | 2.32E-1 | 3.68 (961,174,21,14) |
| *6 h treatment. Downregulated genes.* | | | | |
| GO:0031577 | spindle checkpoint | 8.48E-5 | 5.81E-1 | 5.82 (961,6,165,6) |
| *6 h treatment. Upregulated genes.* | | | | |
| GO:1903035 | negative regulation of response to wounding | 5.45E-6 | 3.74E-2 | 16.15 (961,17,21,6) |
| GO:0032102 | negative regulation of response to external stimulus | 5.7E-6 | 1.95E-2 | 12.32 (961,26,21,7) |
| GO:0051591 | response to cAMP | 2.12E-5 | 4.85E-2 | 68.64 (961,7,6,3) |
| GO:0032501 | multicellular organismal process | 2.33E-5 | 3.98E-2 | 1.30 (961,205,482,134) |
| GO:0050793 | regulation of developmental process | 2.87E-5 | 3.93E-2 | 2.03 (961,188,98,39) |
| GO:0044707 | single-multicellular organism process | 3.3E-5 | 3.77E-2 | 1.30 (961,204,482,133) |
| GO:0007165 | signal transduction | 4.41E-5 | 4.31E-2 | 1.50 (961,354,141,78) |
| GO:0051240 | positive regulation of multicellular organismal process | 8.19E-5 | 7.01E-2 | 1.35 (961,125,517,91) |
| GO:0032101 | regulation of response to external stimulus | 9.77E-5 | 7.44E-2 | 1.50 (961,56,536,47) |
